# Beyond traditional anticoagulants: exploring the future of thromboembolism treatment with FXI inhibitors

**DOI:** 10.3389/fimmu.2026.1925585

**Published:** 2026-10-05

**Authors:** Hui Yuan, Yuanyuan He, Mao Zhang, Qinan Yin

**Affiliations:** 1 School of Medicine, University of Electronic Science and Technology of China, Chengdu, China; 2 Department of Pharmacy, Personalized Drug Research and Therapy Key Laboratory of Sichuan Province, Sichuan Provincial People’s Hospital, School of Medicine, University of Electronic Science and Technology of China, Chengdu, China; 3 Department of Vascular Surgery, Sichuan Academy of Medical Sciences & Sichuan Provincial People’s Hospital, School of Medicine, University of Electronic Science and Technology of China, Chengdu, China

**Keywords:** clinical outcomes, complement, FXI, FXI inhibitors, FXIa, immunothrombosis, NETosis, thromboembolic diseases

## Abstract

Thromboembolic diseases impose a significant burden on global health. However, the currently approved anticoagulant drugs, including heparin, vitamin K antagonists, and direct oral anticoagulants (DOACs), all carry certain bleeding risks. The proteases inhibited by these drugs play crucial roles in both pathological thrombosis formation and normal hemostasis. Therefore, how to effectively anticoagulate without increasing bleeding has become the core direction of clinical research. Factor XI (FXI) and activated factor XI (FXIa) are increasingly recognized as regulators at the interface between coagulation and immunothrombosis. Experimental studies suggest that FXI/FXIa may interact with neutrophil extracellular trap formation (NETosis), complement activation, and inflammatory signaling pathways, raising the possibility that FXI/FXIa inhibition could have broader effects beyond anticoagulation. However, the clinical relevance of these immunomodulatory functions remains to be established. Genetic evidence also supports this characteristic: FXI deficiency can reduce the risk of venous thromboembolism (VTE) and does not lead to spontaneous bleeding, whereas elevated FXI levels increase thrombotic events. Thus, FXI/FXIa have become promising targets due to their unique biological properties. Currently, FXI inhibitors have been developed in various formulations, including antisense oligonucleotides, monoclonal antibodies, and small - molecule synthetic drugs. The phase II clinical trials of FXI inhibitors have achieved positive results in the treatment of knee joint replacement surgery, end - stage renal disease, atrial fibrillation (AF), acute non - cardiogenic thromboembolic ischemic stroke, and high - risk transient ischemic attack (TIA). Phase III clinical trials of certain FXI inhibitors are also being actively conducted. The review focuses on the spatial structure of FXI, its role in the coagulation pathway and pathological thrombosis formation, immunothrombosis and gene polymorphisms. It introduces the current cutting - edge drugs in development, summarizes the results of completed clinical trials, and discusses the current research progress.

## Highlights

- FXI/FXIa separates thrombosis from hemostasis:

FXI/FXIa have emerged as promising anticoagulant targets due to their distinct contribution to pathological thrombosis and physiological hemostasis. Unlike conventional anticoagulants targeting factor Xa or thrombin, FXI inhibitors primarily suppress thrombin amplification during thrombus propagation while largely preserving initial hemostatic responses, providing a potential strategy to reduce bleeding complications.

- FXI/FXIa links coagulation with immunothrombosis:

Beyond coagulation amplification, FXI/FXIa participate in immunothrombotic processes through interactions with neutrophil extracellular traps (NETs), complement regulation, and inflammatory signaling pathways. These findings expand the potential therapeutic scope of FXI/FXIa inhibition beyond conventional anticoagulation, although its clinical relevance in inflammatory diseases requires further investigation.

- Distinct FXI/FXIa-targeting platforms determine therapeutic opportunities:

Oral small-molecule FXIa inhibitors (e.g., Asundexian and Milvexian) provide rapid and reversible inhibition, making them suitable for acute thrombotic settings. Monoclonal antibodies (e.g., Abelacimab and Osocimab) enable sustained FXI/FXIa suppression, whereas ASO/small interfering RNA (siRNA) approaches provide prolonged reduction of FXI synthesis and may be advantageous for long-term prevention strategies.

- Disease-specific FXI dependency determines clinical efficacy:

The clinical benefit of FXI inhibitors is largely determined by the biological dependence of individual thrombotic phenotypes on FXI-mediated coagulation amplification rather than drug class alone. FXI inhibitors have shown consistent antithrombotic efficacy with limited bleeding in postoperative VTE prevention, whereas results in atrial fibrillation (AF) suggest that FXI inhibitors alone may be insufficient for thrombotic conditions driven predominantly by FXI-independent pathways.

- OCEANIC-STROKE supports FXIa inhibitors as a complementary strategy:

The phase III OCEANIC-STROKE trial demonstrated that adding Asundexian (50 mg once daily) to antiplatelet therapy reduced ischemic stroke risk without increasing major bleeding. These findings support FXIa inhibitors as a complementary approach to suppress residual coagulation-driven thrombus propagation in atherothrombotic disease rather than a replacement for antiplatelet therapy.

## Introduction

In recent years, thromboembolic diseases are primarily categorized as arterial thromboembolism (ATE) and venous thromboembolism (VTE). ATE mainly manifests as ischemic heart disease and ischemic stroke. These conditions account for one - quarter of global deaths and are established as a major worldwide health concern (1). Among these, ischemic heart disease (including post-myocardial infarction, angina, or heart failure) affected an estimated 8.99 million individuals (95% uncertainty interval (UI) 8.29–9.55), with approximately 188 million disability-adjusted life years (DALYs) (95% UI 177–198). Approximately 7.81 million (6.72–8.94) individuals suffered from ischemic stroke, with DALYs estimated at 70.4 million (95% UI 64.1–76.1) and deaths at around 350,000 (95% UI 322–389). DALYs attributable to atrial fibrillation (AF) and flutter amounted to 8.36 million (95% UI 6.97 million–10.10 million), representing a 148.9% increase (95% UI 134.2%–166.6%) compared to 1990. The number of deaths attributable to AF and atrial flutter was 339,000 (95% UI 286,000–368,000) (2).

VTE encompasses deep vein thrombosis (DVT) and pulmonary embolism (PE). With an estimated annual incidence of 10 million cases, it ranks as the third most prevalent vascular disease following acute myocardial infarction (AMI) and stroke. In six European countries with a combined population of 300 million, approximately 500,000 cases of DVT and 300,000 cases of PE are reported annually. In the United States, the economic impact of VTE is considerable, with annual costs estimated between $7 billion and $10 billion. The incidence of VTE is on the rise, attributed to factors such as an aging population, an increased prevalence of comorbidities associated with VTE—including obesity, heart failure, and cancer—and advancements in diagnostic test sensitivity (3). The estimated incidence of VTE ranged from 79 per 100,000 (4) to 269 per 100,000 (5). Anticoagulant therapy remains a cornerstone in the treatment of thromboembolic disorders, effectively suppressing both thrombus formation and extension.

Over the past few decades, anticoagulant therapy has undergone significant advancements. The early treatment regimens were mainly based on heparin and vitamin K antagonists (VKAs). Subsequently, direct oral anticoagulants (DOACs) came into being, offering advantages such as user-friendliness, convenience, reduced interactions with food or other medications, a diminished need for frequent coagulation monitoring, improved compliance, and a relatively lower risk of bleeding. They are now widely employed in the prevention and treatment of various thromboembolic diseases. However, DOACs primarily function by directly or indirectly inhibiting activated factor X (FXa) and/or thrombin. These proteases play crucial roles in overall hemostasis. Inhibiting their functions compromises normal clotting mechanisms and elevates bleeding risks, which include gastrointestinal hemorrhage and intracranial hemorrhage. Furthermore, DOACs face limitations, including the absence of specific antidotes and restricted use in patients with chronic kidney disease (6).

Studies in individuals with a history of cardiovascular disease or an increased risk indicate that elevated Factor XI (FXI) levels correlate with a heightened probability of stroke, transient ischemic attack(TIA), myocardial infarction (MI), and severe coronary artery disease, suggesting that high FXI levels increase the thrombotic risk (7, 8). Individuals with elevated FXI levels face higher venous thromboembolism risk than the general population (9), while those with FXI deficiency show lower DVT rates and rarely experience spontaneous bleeding, with plasma FXI levels having no impact on bleeding probability (10). Additionally, multiple animal models have demonstrated that FXI inhibitors possess potent antithrombotic effects without marked bleeding propensity (11). FXI/FXIa inhibition can separate the antithrombotic effect of anticoagulant therapy from the risk of bleeding. This favorable profile arises from the distinct roles of FXI/FXIa in pathological thrombosis versus physiological hemostasis: FXI/FXIa play pivotal roles in the propagation of pathological thrombi, whereas it contributes only marginally to physiological clot stabilization and exerts a secondary function in normal hemostasis. Given its unique biological role, FXI/FXIa are increasingly recognized as key targets for the development of safer anticoagulant agents (12).

Currently, several classes of FXI inhibitors have been developed, including antisense oligonucleotides (ASO) that suppress FXI biosynthesis, monoclonal antibodies that prevent zymogen activation, and small - molecule synthetics, natural peptides, and aptamers that directly inhibit the activated form of FXI. Multiple clinical trials are underway or in the planning stages to define the clinical utility of FXI inhibitors and to identify the most appropriate inhibitor modality for specific indications. This article first analyzes the spatial structure of FXI, and then systematically explores its function in the coagulation cascade reaction and the mechanism of its involvement in pathological thrombosis formation. It focuses on summarizing the research progress of new FXI inhibitors based on chemical modification strategies, and discusses the current progress and future challenges, with the aim of providing new ideas for optimizing safer anticoagulation strategies.

## Structure of FXI

Among various coagulation factors, FXI possesses a unique evolutionary origin, derived from the duplication of the kallikrein B1 (KLKB1) gene encoding plasma prekallikrein (PK), the zymogen form of plasma kallikrein (PKa), the enzyme that releases bradykinin (BK) from high molecular weight kininogen (HK). This evolutionary background endows FXI with a unique homodimeric structure (molecular weight approximately 160 kDa). As shown in **Figure 1**, the FXI molecule exhibits a distinctive ‘cup-and-saucer’ spatial conformation, consisting of two identical subunits each containing 607 amino acids. Each subunit comprises four apple-like domains at the N-terminus (A_1_-A_4_, each containing 90 or 91 amino acids) and a catalytic domain (light chain) at the C-terminus (13, 14). Each apple domain is composed of seven β-strands forming an antiparallel β-sheet, supporting a central α-helix that is anchored to the concave surface of the sheet via a disulfide bond (15). The four apple domains have distinct binding functions: thrombin and HK bind to A1 (16, 17); A2 interacts with HK (18), A3 acts on factor IX (FIX) (19), platelet receptor glycoprotein Ib (GPIb (20) and heparin (21, 22). At the same time, A4 engages activated factor XII (FXIIa) and other FXI subunits (15, 23). The four apple domains collectively form a disc-like structure. The catalytic domain, also known as the light chain, sits atop this platform, forming a “cup-and-saucer” configuration that facilitates FXI activation. The light chain contains the active site of activated factor XI (FXIa), which exerts its function by cleaving various substrates, a process mediated by the A1 to A4 domains of the heavy chain. Under the coordination of the A3 domain, FIX is sequentially cleaved by the protease domain of FXIa at residues Arg145 - Val146 and Arg180 - Val181, ultimately generating the fully activated form of FIX, FIXa. The FXI dimer is stabilized by an interchain disulfide bond between Cys321 residues in the A4 finger loops, along with a hydrophobic interface (Leu284, Ile290, Tyr329) and an interchain salt bridge (Lys331–Glu287) (15, 24, 25). From a physiological perspective, FXI is activated through the cleavage of the Arg369-Ile370 bond by either thrombin or FXIIa. Additionally, FXI can undergo self-activation in the presence of polyanions, including inorganic polyphosphate polymers. Under physiological conditions, plasma serine protease inhibitors—such as antithrombin, α1-antitrypsin, C1 inhibitors, and protein Z-dependent protease inhibitors—may inhibit FXIa. Furthermore, sulfated glycosaminoglycans, such as heparin, can also inhibit the physiological function of FXIa (26). These domains exhibit varying affinities for binding partners within the coagulation cascade, thereby influencing both the activation of FXI to its serine protease form and the specificity of substrates cleaved by FXIa.

**Figure 1:**
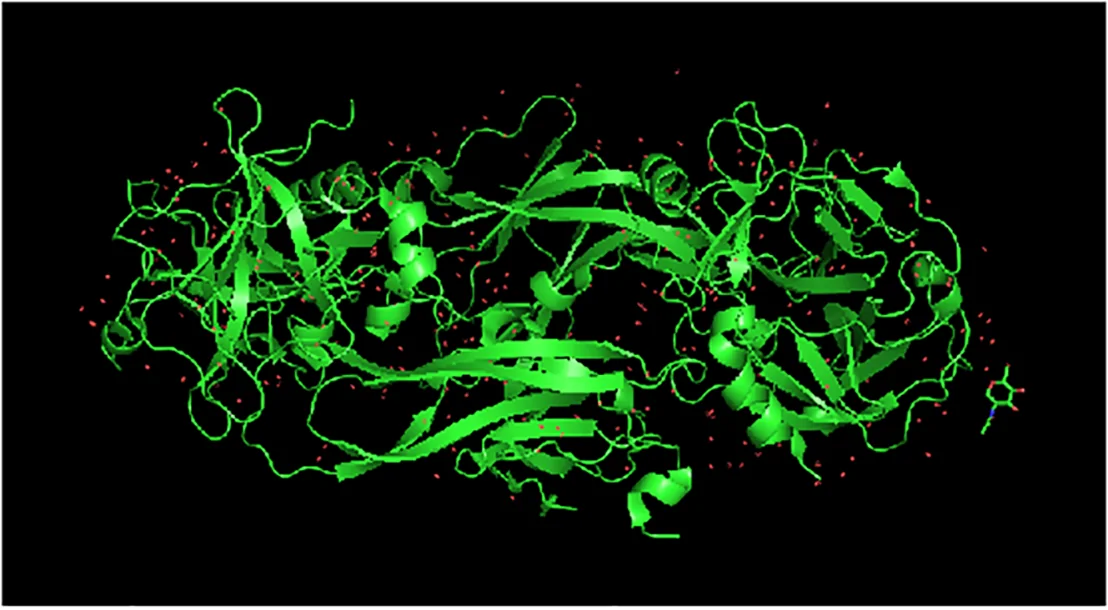
Spatial structure of FXI (The protein sequence of FXI comes from Nucleic Acids Res. 53:D609-D617(2025), and was then exported using PYMOL. https://www.uniprot.org/uniprotkb/P03951/external-links).

## The mechanism of FXI in thrombosis

### The central prothrombotic mechanism of FXI

FXI primarily participates in the intrinsic coagulation pathway but also cross-regulates the extrinsic pathway. During the initiation phase of thrombosis, the extrinsic pathway rapidly generates a small amount of thrombin, which in turn can activate FXI, establishing a robust positive feedback amplification loop. This loop drives the substantial thrombin burst that potentiates thrombus formation, explaining why patients with severe congenital FXI deficiency typically exhibit only a mild bleeding tendency (27). Studies have found that FXIa can hydrolyze tissue factor pathway inhibitor (TFPI), thereby increasing the procoagulant activity of the extrinsic pathway (28). Polyphosphate (polyP) released from platelets can accelerate this process (29). Additionally, FXIa can inactivate a disintegrin and metalloproteinase with thrombospondin type 1 motif, member 13 (ADAMTS13), a key metalloprotease that limits platelet recruitment by cleaving the adhesive protein von Willebrand factor (vWF) (30). During the thrombus enlargement stage, FXIIa activates FXI to generate FXIa, which in turn activates FIX to produce FIXa. FIXa and its cofactor activated factor VIII (FVIIIa) form an endogenous thrombin complex, which efficiently converts factor X (FX) to activated factor X (FXa), thereby promoting thrombin production. Thrombin itself also can activate FXI, creating a positive feedback loop that results in a cascade of thrombin generation, thereby powerfully driving the amplification of the coagulation process (31, 32). Moreover, FXIa can directly activate factors X, V and VIII (FX, FV, FVIII) in a calcium-independent manner, further promoting thrombin generation (33, 34). Platelet release products, including polyphosphate, can significantly enhance the feedback activation of FXI by thrombin and accelerate the activation of FV by FXIa, in a manner analogous to polyP (29, 35). During the stabilization phase, FXIa does not directly inhibit the fibrinolytic system. However, the excess thrombin generated via its procoagulant action, upon reaching a critical threshold, activates thrombin-activatable fibrinolysis inhibitor (TAFI). Activated TAFI protects the fibrin clot from degradation by the fibrinolytic system, thereby stabilizing the formed thrombus. This represents a key mechanism by which FXI contributes to thrombus stability (36).

### FXI/FXIa-mediated crosstalk among inflammation, complement, and immunity

Notably, FXI/FXIa involved not only in coagulation but also cross-regulates inflammation, complement, and immunity. The recently proposed concept of “immunothrombosis” (37) refers to thrombosis mediated by interactions between infection-activated neutrophils/monocytes, activated platelets, and coagulation factors. These thrombi serve a protective function by limiting, trapping, and clearing pathogens and can occur in arteries, veins, and microvessels (38). The basic principles of immunothrombosis include: upon microbial invasion, their products induce the synthesis and release of inflammatory mediators known as pathogen-associated molecular patterns. The damage-associated molecular patterns (DAMPs) released by activated or damaged cells also have pro-inflammatory effects; monocytes and their microvesicles express and deliver activated tissue factors to the site of pathogen exposure, initiating the extrinsic pathway (39, 40). A key node in immunothrombosis is neutrophils and neutrophil extracellular traps (NETs). The NETs released by activated neutrophils, with their DNA and histone backbones carrying a strong negative charge, provide an ideal surface for contact system activation. This effectively activates FXII, which in turn activates FXI, thereby activating the entire coagulation system (41). DAMPs activation causes platelets to release high mobility group protein B1 (HMGB-1) and calprotectin, which stimulates the formation of NETs (42, 43). Therefore, activation of the coagulation cascade involving FXI can indirectly promote NETs release through downstream products and cellular crosstalk, forming a positive feedback loop. FXIa can inhibit the chemotaxis of human polymorphonuclear leukocytes toward exogenous chemokines, which may promote neutrophil retention at the site of coagulation initiation rather than migration to distant areas, thereby facilitating local immune cell aggregation and inflammation (44). Activated neutrophils can also release neutrophil gelatinase-associated lipocalin, which can significantly enhance FXIa enzymatic activity and upregulate the expression and activity of cell surface tissue factor (45). Furthermore, NETs provide a scaffold for blood cells and procoagulant factors, accelerating thrombin generation and fibrin formation, thereby enhancing their vascular occlusive effect (42).

FXI/FXIa also amplifies the inflammatory response through cross-regulation of the complement system. FXIa specifically cleaves the complement control protein domain 6 (CCP6) domain of complement factor H (CFH), resulting in the inactivation of CFH, inhibiting the degradation of the C3 convertase in the alternative pathway, causing an increase in C3b levels and triggering excessive activation of the alternative pathway (46, 47). Complement overactivation produces the anaphylatoxins C3a and C5a, which are potent inflammatory mediators that strongly recruit neutrophils and monocytes, and activate endothelial cells and platelets, forming a powerful pro-inflammatory and pro-thrombotic feedback loop (48). C5a activates neutrophils through complement C5a receptor 1 (C5aR1), causing them to overexpress tissue factor and interact with platelets, thereby releasing NETs containing tissue factor. NETs provide a procoagulant surface and feedback-activate the contact system. Tissue factor (TF) initiates the extrinsic coagulation pathway, participating in immunothrombosis (49). FXI/FXIa can also bind to the complement positive regulator protein properdin, which may further regulate the complement activity (50).

Beyond its role in coagulation, Experimental studies have shown that FXI deficiency or inhibition attenuates the inflammatory response, including alterations in the production of early pro-inflammatory cytokines such as tumor necrosis factor-α (TNF-α) and interleukin-6 (IL-6) (51). Within the contact system, however, the canonical reciprocal activation of FXII and the release of BK from HK are primarily mediated by PKa, rather than representing major functions of FXIa. FXIIa activates PK to generate PKa, which in turn promotes further the activation of FXII and cleaves HK to release BK, thereby amplifying inflammatory signaling (52, 53). Although FXIa can cleave HK *in vitro*, this cleavage is mainly associated with loss of the surface-binding and cofactor functions of HK and may contribute to the regulation of contact-system activation rather than to the canonical generation of BK (54). In addition to these indirect effects, FXI/FXIa can directly modulate immune and inflammatory pathways through proteolytic processing of specific substrates. FXIa hydrolyzes the inactive precursor chemokine in a dose-dependent manner, generating the active chemokine. This active chemokine acts on dendritic cells, macrophages, etc., through its receptor chemerin chemokine-like receptor 1 (CMKLR1), establishing a direct molecular bridge between coagulation and innate/adaptive immunity (55). FXI/FXIa can also regulate inflammatory responses by cleaving other inflammatory proteins, including β2-glycoprotein I and prochemerin (56, 57).

FXI/FXIa serve as important links between coagulation and immunothrombosis. Although experimental evidence suggests that FXI/FXIa inhibition may influence neutrophil extracellular trap formation (NETosis), complement activation, and inflammatory pathways, these immunomodulatory effects remain to be clinically validated. Future studies are required to determine whether FXI inhibitors can provide therapeutic benefits in immunothrombotic disorders beyond their established anticoagulant effects.

### Genetic polymorphisms of FXI and their clinical associations

At the genetic level, substantial evidence links FXI gene polymorphisms to thrombotic susceptibility. Two FXI SNPs, rs2289252 and rs2036914, are independently associated with the risk of DVT, and their risk alleles are significantly correlated with elevated plasma FXI levels. After correcting the FXI level, the association still exists but has weakened. This suggests that its impact on the risk of DVT is at least partially achieved by regulating the FXI level (58). A prospective cohort study involving 1,010 women with a first episode of VTE found that the FXI rs2289252 SNP increased the risk of VTE recurrence. The cumulative recurrence rate was 2.5 times higher in carriers of FXI rs2289252 who also carried the FV rs6025 (Factor V Leiden) mutation compared to non-carriers (59). In post-traumatic fracture patients, the FXI rs2289252 T and rs2036914 C alleles are associated with higher plasma FXI activity, a correlation that persists despite conventional thromboprophylaxis. High FXI activity is a significant risk factor for DVT in these patients despite preventive anticoagulation (60). Beyond common SNPs, an FXI gene duplication has also been reported to elevate FXI levels and correlate with venous thrombotic risk in a family (61). Conversely, FXI deficiency is associated with a reduced incidence of cardiovascular events and VTE (62). Regarding arterial thrombosis, existing evidence does not robustly support a clear association between FXI and arterial thrombotic events. The SNP loci rs925451, rs3733403 and rs1593 may be associated with ischemic stroke in individuals aged 70 and below. Among them, rs925451 still maintains a relatively weak association in the repeated cohort (≤ 70 years) (63). Mendelian randomization studies suggest that FXI is a potential therapeutic target for reducing the risk of ischemic stroke, particularly the cardioembolic subtype, but its association with myocardial infarction and intracerebral hemorrhage is not evident (64).

Evidence concerning the association between FXI gene polymorphisms and drug efficacy remains primarily exploratory. The MEGA study suggests that statin use may attenuate the venous thrombotic risk conferred by the rs2289252/rs2036914 risk alleles, although the underlying mechanism is not yet clear (65). In percutaneous coronary intervention (PCI) patients, the FXI rs2036914 CC genotype is associated with a higher risk of PCI-related myocardial infarction, while the T allele is linked to a higher risk of minor bleeding (66). Currently, robust evidence for guiding clinical medication based on FXI genotypes is lacking. However, in-depth studies on the polymorphism of FXI have revealed key genetic pathways in the pathogenesis of thrombosis, which is helpful for risk stratification, optimizing prevention strategies, individualized treatment.

Overall, FXI/FXIa occupies a pivotal node in the coagulation, inflammatory, and complement systems. FXI/FXIa serves not only as a key amplifying factor in the intrinsic coagulation pathway but also plays a profound role in immunothrombosis and inflammatory responses (**Figure 2**).

**Figure 2:**
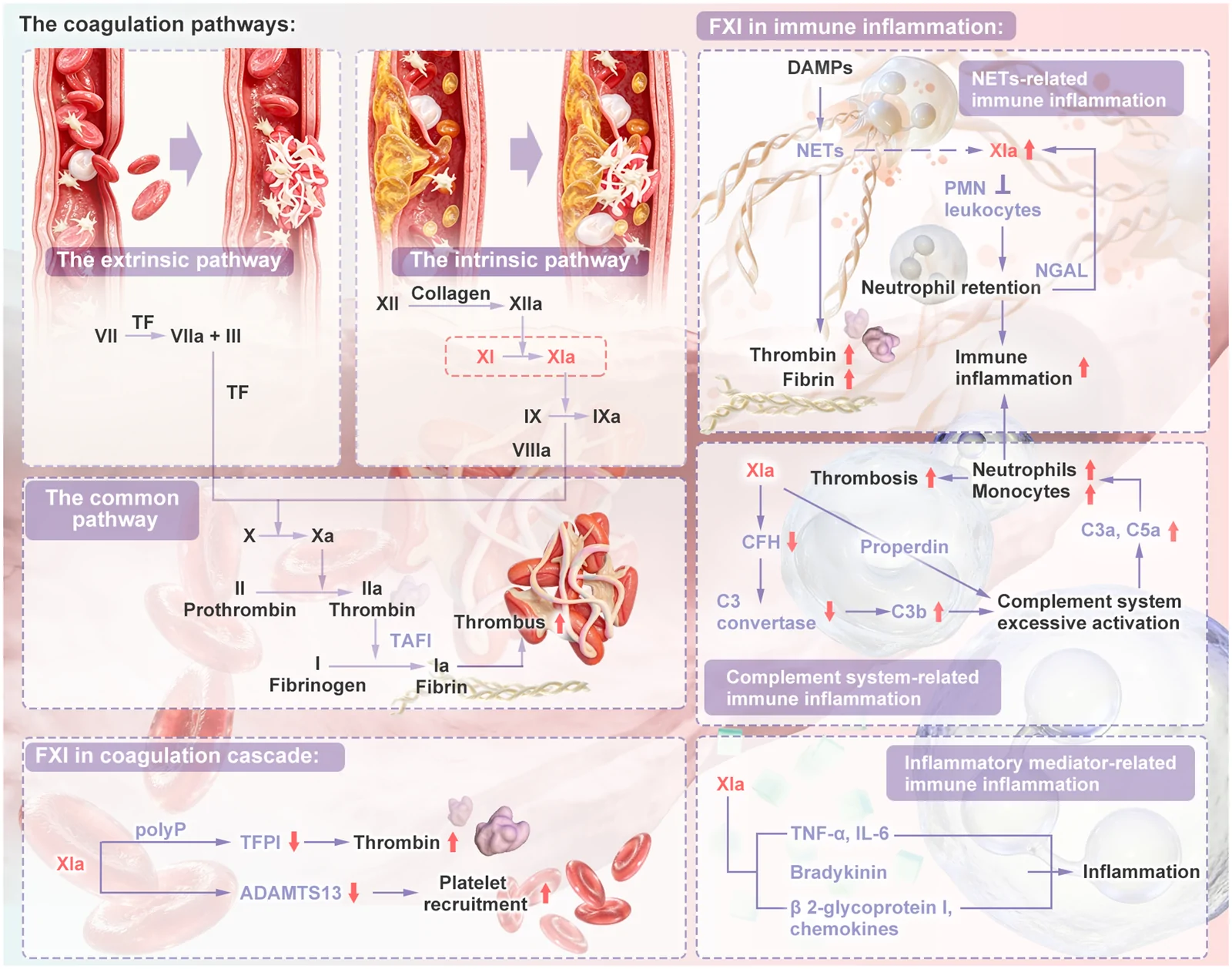
The specific role of FXI/FXIa in the coagulation pathway and the mechanisms involved in immunothrombosis and inflammation. ADAMTS13: A Disintegrin And Metalloproteinase With A Thrombospondin Type 1 Motif, Member 13; CFH: Complement Factor H; C3 convertase: Complement Component 3 Convertase; C3a: Complement Component 3a; C3b: Complement Component 3b; C5a: Complement Component 5a; DAMPs: Damage-Associated Molecular Patterns; FI/FIa: Fibrinogen/Fibrin; FII/FIIa: Prothrombin/Thrombin; FVII/FVIIa: Factor VII/Activated Factor VII; FVIII/FVIIIa: Factor VIII/Activated Factor VIII; FIX/FIXa: Factor IX/Activated Factor IX; FX/FXa: Factor X/Activated Factor X; FXI/FXIa: Factor XI/Activated Factor XI; FXII/FXIIa: Factor XII/Activated Factor XII; FXIII: Factor XIII; TF: Tissue Factor; TFPI: Tissue Factor Pathway Inhibitor; TAFI: Thrombin-Activatable Fibrinolysis Inhibitor; polyP: Polyphosphate; IL-6: Interleukin-6; NETs: Neutrophil Extracellular Traps; NGAL: Neutrophil Gelatinase-Associated Lipocalin; PMN: Polymorphonuclear Leukocytes.

## FXI/FXIa-targeted anticoagulants

Factor II (FII) and FXI/FXIa have been identified as promising therapeutic targets for the prevention of VTE and cardioembolic stroke (CES), with a favorable safety profile characterized by a low bleeding risk, as supported by a genome-wide association meta-analysis integrating Mendelian randomization and genetic co-localization proteomics data from two independent cohorts (67). Among these, FXI/FXIa play a pivotal role in pathological thrombosis while contributing only marginally to physiological hemostasis. This dissociable functionality renders it an attractive target for anticoagulant development, with the potential to achieve potent antithrombotic efficacy without substantially increasing bleeding complications. Based on the distinct stages of FXI/FXIa activation and function in thrombosis (**Figure 3**), current pharmacological strategies can be categorized as follows:1. Reduction of FXI protein synthesis – This approach inhibits FXI production at the transcriptional or translational level. For instance, FXI-directed ASOs hybridize with FXI mRNA through sequence-specific interactions, while siRNA molecules facilitate mRNA degradation via RNA-induced silencing complex (RISC)-mediated cleavage, both leading to suppressed FXI expression.2.Prevention of FXI activation – Monoclonal antibodies such as Abelacimab and Osocimab bind to specific domains of FXI (e.g., the catalytic or apple domains), sterically hindering its proteolytic activation and consequent conformational changes required for activity.3.Direct inhibition of FXIa enzymatic activity – Small-molecule inhibitors including Asundexian and Milvexian bind reversibly or irreversibly to the active site of FXIa, competitively blocking substrate recognition and hydrolysis.4.Interference with FXIa–substrate/cofactor interactions – Agents such as certain monoclonal antibodies and aptamers do not target the catalytic center of FXIa but instead disrupt its binding to downstream substrates (e.g., FIX) or cofactors (e.g., polyanionic surfaces on platelets), thereby attenuating amplification of the coagulation cascade. Currently, the development of FXI inhibitors has encompassed various technological approaches, including small molecule inhibitors, monoclonal antibodies, ASOs and siRNA. **Table 1** summarizes key information on the major novel FXI inhibitors under development, including their target molecules, development stages, clinical characteristics, and outcomes from preclinical and clinical trials.

**Figure 3:**
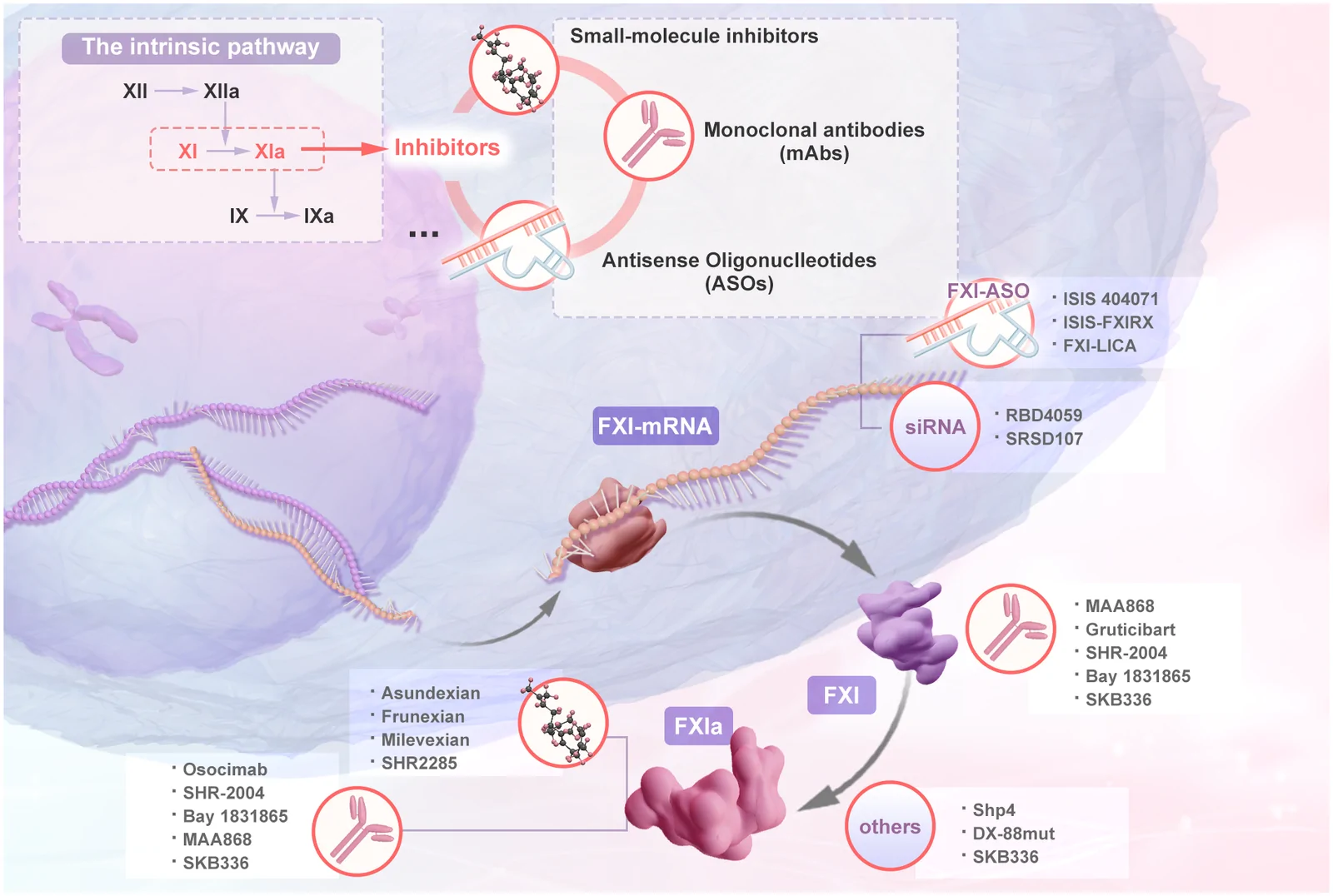
The target site of the new FXI inhibitors.

**Table 1:** Representative factor XI(FXI) inhibitors in phase III trials.

Drug name	Classification	All indications	Number of participants	Trial ID	Key findings	Dose	Clinical trial status	Reference
Asundexian	small molecule	Secondary Stroke Prevention	12327	NCT05686070	Among patients with noncardioembolic ischemic stroke or high-risk TIA treated with antiplatelet therapy, Asundexian at a daily dose of 50 mg resulted in lower risks of ischemic stroke and major cardiovascular events than placebo, without a higher risk of major bleeding.	Oral 50 mg once daily	Completed	(68)
		Stroke Prevention in AF	14810	NCT05643573	Among patients with atrial fibrillation at risk for stroke, treatment with asun dexian at a dose of 50 mg once daily was associated with a higher incidence of stroke or systemic embolism than treatment with apixaban in the period before the trial was stopped prematurely. There were fewer major bleeding events with Asundexian than with apixaban during this time		Early Termination	(69)
Milvexian	small molecule	AF	20284(Submitted:2026-02-12)	NCT05757869	The purpose of this study is to evaluate if Milvexian is at least as effective as apixaban for reducing the risk of the composite stroke and non-central nervous system (CNS) systemic embolism	Oral 100 mg twice daily	Ongoing	/
		Acute Coronary Syndrome	14194(Submitted: 2025-12-19)	NCT05754957	The purpose of this study is to evaluate that Milvexian is superior to placebo, in addition to standard-of-care, in reducing the risk of major adverse cardiovascular event (MACE) (the composite of cardiovascular [CV] death, myocardial infarction [MI], and ischemic stroke).	Oral 25mg twice daily	Completed	/
		Acute Ischemic Stroke or High-Risk Transient Ischemic Attack	12532(Submitted: 2026-05-07)	NCT05702034	The purpose of this study is to evaluate whether Milvexian compared to placebo reduce the risk of recurrent ischemic stroke.	Oral 25mg twice daily	Ongoing	/
Abelacimab	monoclonal antibody	AF	1900(Estimated)	NCT05712200	A study to evaluate the effect of abelacimab relative to placebo on the rate of ischemic stroke or systemic embolism (SE) in patients with Atrial Fibrillation (AF) who have been deemed by their responsible physicians or by their own decision to be unsuitable for oral anticoagulation therapy.	150mg intravenous	Ongoing	/
		Cancer-associated VTE	1150(Submitted: 2025-08-27)	NCT05171049	This is a Phase 3,multicenter, randomized, open-label, blinded endpoint evaluation study comparing the effect of abelacimab relative to apixaban on venous thromboembolism (VTE) recurrence and bleeding in patients with cancer associated VTE (ASTER)	150mg intravenous	Completed	/
		Treatment of Gastrointestinal/​Genitourinary Cancer and Associated VTE	417(Submitted: 2025-12-18)	NCT05171075	To evaluate the effect of abelacimab relative to dalteparin on venous thromboembolism (VTE) recurrence and bleeding in patients with gastrointestinal (GI)/genitourinary (GU) cancer associated VTE (Magnolia)	150mg intravenous	Completed	/
SHR-2004	monoclonal antibody	Preventing Venous Thromboembolism in Patients Undergoing Total Knee Arthroplasty	1166(Submitted: 2026-03-09)	NCT06825416	The purpose of the study is to evaluate the efficacy and safety of SHR-2004 in preventing venous thromboembolism after elective unilateral total knee arthroplasty.	90 mg intravenous	Completed	/.

## Direct inhibition of FXIa enzymatic activity: opportunities and challenges for oral small-molecule inhibitors

The indications for oral FXIa small-molecule inhibitors show significant divergence. They demonstrate the strong translational potential in the secondary prevention of atherothrombotic events (especially non-CES and TIA), while facing major challenges in stroke prevention for AF. They also exhibit competitiveness in the field of venous thrombosis prophylaxis.

Asundexian is an oral reversible FXIa inhibitor that has been extensively evaluated across several thrombotic conditions. Early phase studies demonstrated potent FXIa inhibition with a favorable bleeding profile (70, 71). In patients with AMI and non-cardioembolic ischemic stroke, compared with the antiplatelet control group, Asundexian combined with antiplatelet therapy effectively inhibited FXIa activity without significantly increasing bleeding, supporting further clinical evaluation (72, 73). Early anticoagulation therapy in patients with ischemic stroke receiving Asundexian in addition to antiplatelet therapy was not associated with increased intracranial hemorrhage and could prevented thrombosis without increasing bleeding, providing favorable evidence for subsequent clinical trials (74). By analysis of cerebral microbleeds, patients receiving Asundexian exhibited lower rates of new microbleeds and intracranial hemorrhage compared to placebo (standard antiplatelet therapy). This indicates that for patients with ischemic stroke in non-core areas and cerebral microvascular disease (CMVD) with microbleeds, Asundexian may be safer (75). And it revealed that Asundexian may prevent new embolic infarcts but does not prevent small artery occlusions (76). Though further studies are required to confirm these conclusions. For patients with acute non-cardiogenic ischemic stroke or high-risk TIA, a phase III clinical trial (OCEANIC-STROKE) involving 12,327 participants evaluated the efficacy and safety of Asundexian combined with antiplatelet therapy in preventing secondary stroke. The results showed that the incidence of ischemic stroke in the Asundexian (50mg•d^-1^) was lower than that in the placebo (6.2% *vs.* 8.4%; HR 0.74; 95% CI 0.65-0.84), and the incidence of cardiovascular death, MI, or stroke-related adverse events was also lower. The rates of major bleeding in both groups were similar. This suggests that for patients with acute non-cardiogenic ischemic stroke or high-risk TIA who are receiving antiplatelet therapy, adding Asundexian is a potential combined treatment option (68).

However, the research results of Asundexian regarding the prevention of thrombosis in patients with AF were different. Although a phase II trial demonstrated that the FXIa inhibitor asundexian, at daily doses of 20 mg and 50 mg, achieved nearly complete *in vivo* FXIa inhibition and was associated with lower bleeding rates compared with standard-dose apixaban in patients with AF (77). However, an international multicenter, phase III, double-blind trial evaluating Asundexian’s ability to prevent thrombosis in patients with AF was prematurely terminated. The study showed that compared to apixaban, Asundexian(50mg•d^-1^) resulted in a higher incidence of stroke or systemic embolism (apixaban hazard ratio(HR) 0.32; 95% confidence interval(CI) 0.18-0.55), but before the trial was prematurely terminated, the incidence of severe bleeding events caused by Asundexian was slightly lower (69). Another trial analysis showed that compared with apixaban, patients with AF who had no history of oral anticoagulant (OAC) and received Asundexian treatment had a lower risk of stroke and systemic embolism (HR 1.42; 95% CI 0.54 - 3.73). However, in patients who had received OAC treatment for more than 6 weeks previously, Asundexian did not show an advantage (Asundexian *vs.* apixaban: 1.4% *vs.* 0.3%), and the specific mechanism causing this difference is still unclear (78).

Frunexian (EP-7041) is a potent and selective FXIa inhibitor for parenteral administration. It has the characteristics of a short half-life, low distribution volume, and low dependence on renal clearance. In a rat mesenteric artery puncture model, there was no statistically significant difference in bleeding risk between the Frunexian group and the heparin group. In a canine model of venovenous extracorporeal membrane oxygenation, bleeding volume in heparin-anticoagulated animals was at least fivefold greater than that observed with the highest tested dose of Frunexian (79). An Phase I study on healthy Chinese volunteers (continuous infusion with increasing doses of 0.3–2.25 mg/kg/h for a total of 5 days) showed that Frunexian was safe, without bleeding events. The activated partial thromboplastin time (APTT) was dose-dependently prolonged, and FXIa activity decreased. The pharmacokinetic/pharmacodynamic characteristics supported its potential use as an anticoagulant, suggesting it may represent a promising anticoagulant agent (80).

Milvexian (BMS-986177/JNJ-70033093) is a highly potent and selective inhibitor of FXIa (81, 82). *In vitro* thrombin assays demonstrated that Milvexian prolonged the APTT in human and rabbit plasma without altering prothrombin time (PT). Its combination with aspirin had minimal effects on coagulation function (83).

In healthy volunteers, Milvexian exhibited dose-proportional pharmacokinetics and pharmacodynamics across different ethnic populations. In Japanese subjects, doses up to 500 mg/day were well tolerated, with dose-dependent increases in plasma concentration and APTT prolongation, achieving up to 74.1% inhibition of FXIa activity at 50–200 mg (8). In Chinese subjects, single or twice-daily doses of 25–200 mg resulted in dose-dependent increases in Maximum Concentration(Cmax) and Area Under the Curve(AUC), a half-life of 9–10 hours, steady state within 6 days, and positive correlation between APTT prolongation and dose/exposure; only mild, clinically insignificant adverse events were reported (84). In Western healthy adults, single doses of 4–500 mg gave a Time to reach Maximum Concentration (Tmax) of 3 hours and half-life of 8.3–13.8 hours; absorption saturated above 300 mg, and food enhanced bioavailability. For multiple dosing, once-daily and twice-daily regimens reached steady state on day 6 and day 3, respectively. Absolute bioavailability studies of spray-dried dispersion (SDD) formulations showed that under fasting conditions, oral solution, 25 mg SDD, and 200 mg SDD had F values of 100%, 58.2%, and 54.2%; after meals, F decreased to 44.3% for 25 mg SDD but increased to 75.6% for 200 mg SDD (85), indicating that the effect of food on bioavailability requires further clarification. Milvexian was well tolerated in patients with mild-to-moderate hepatic impairment (no dose adjustment needed) (86) and in patients with normal renal function or varying degrees of renal impairment (including severe) at 60mg (87). Coadministration with aspirin and/or clopidogrel (200 mg twice daily for 5 days) was safe, without increased bleeding risk or altered Milvexian plasma levels (88). Comprehensive analysis confirmed that concomitant antiplatelet therapy did not affect bleeding time, or platelet aggregation (89). Milvexian shows promising safety, tolerability, and Pharmacokinetics/Pharmacodynamics profiles in patients with hepatic or renal dysfunction, supporting further clinical development. However, the influence of food intake on its bioavailability remains to be fully elucidated.

In a parallel Phase II trial involving 1,242 patients undergoing knee replacement surgery, subjects received one of seven postoperative regimens of Milvexian (25 mg, 50 mg, 100 mg, or 200 mg twice daily, or 25 mg, 50 mg, or 200 mg once daily) or enoxaparin (40 mg once daily). Results demonstrated significantly lower VTE rates in patients receiving twice-daily Milvexian and single 200mg doses compared to the heparin group, with no significant difference in bleeding events between Milvexian and heparin groups. This trial further supports the efficacy of postoperative oral Milvexian in preventing VTE, with a relatively low risk of bleeding (90). A previous phase II randomized trial involving 367 centers in 27 countries randomly assigned patients aged ≥ 40 years with acute (< 48 hours) ischemic stroke or high-risk TIA to Milvexian (one of five regimens: 25 mg QD, 25 mg BID, 50 mg BID, 100 mg BID, or 200 mg BID) or the placebo group for 90 days, combined with aspirin (100 mg for 90 days) and clopidogrel (75 mg for the first 21 days). The results showed that Milvexian combined with dual antiplatelet therapy did not significantly reduce the composite endpoint of symptomatic ischemic stroke or occult cerebral infarction, nor did it significantly increase the risk of major bleeding. This provided a basis for subsequent phase III studies (91). Based on this, a phase III randomized, double-blind, placebo-controlled trial (NCT05702034) is currently evaluating the efficacy and safety of Milvexian in preventing ischemic stroke after acute ischemic stroke or high-risk TIA. Additionally, the ongoing LIBREXIA AF trial (NCT05757869) is a global Phase III randomized double-blind, placebo-controlled trial that compares the efficacy of Milvexian and apixaban in patients with AF/atrial flutter. Another phase III Librexia ACS trial (NCT05754957) for recent acute coronary syndrome (ACS) was prematurely terminated by the independent data monitoring committee due to a pre-specified interim analysis indicating that it was unlikely to achieve the primary efficacy endpoint, but no new safety issues were observed.

SHR2285 (BMS-962212) is a small-molecule inhibitor targeting FXIa. Preclinical studies confirmed its selective inhibition of human FXIa activity, antithrombotic effect, prolongation of APTT, and reduced bleeding risk (92). Pharmacodynamically, SHR2285 produced exposure-dependent prolongation of APTT and reduction of FXIa activity. At steady state, the geometric mean maximum inhibition rates of FXIa activity in the 100–400 mg groups were 73.27%, 85.58%, 87.77%, and 86.27%, respectively. Pharmacological activity tended toward saturation in the 200–400 mg range, without affecting PT or international normalized ratio (INR) (93–95). Pharmacokinetically, after single-dose administration, the median Tmax was 1.50–3.00 h, half-life was 8.74–12.1 h, and total exposure of the metabolite SHR164471 was 1.77–3.61 times that of the parent drug; steady state was reached after 6 days of repeated dosing, with a low accumulation ratio (0.956–1.56).

Multiple studies in healthy volunteers demonstrated good tolerability, with no clinically significant bleeding and only mild adverse events. Coadministration with aspirin, clopidogrel, or ticagrelor was well tolerated, with no increased bleeding risk; the half-life was approximately 14 h, and significant FXIa inhibition and APTT prolongation were maintained (96). Overall, SHR2285 demonstrated good tolerability, predictable pharmacokinetic characteristics, and exposure-related pharmacodynamic properties, establishing a foundation for subsequent clinical trials.

## Blocking FXI activation: the rise of long-acting monoclonal antibodies

Monoclonal antibodies demonstrate advantages in AF and postoperative prophylaxis, including long-acting efficacy, noninferiority, and an exceptionally low bleeding risk, and effectively address the challenge of high bleeding risk. Concurrently, they are actively being explored in historically challenging anticoagulation settings such as end-stage renal disease (ESRD) and cancer, showcasing their potential to address complex clinical needs. In contrast to oral small-molecule inhibitors, which primarily target atherothrombotic events, the development of monoclonal antibodies focuses more on safe anticoagulation in the context of the venous system and cardioembolic conditions.

Abelacimab (MAA868) is a humanized antibody that binds to the catalytic domain of both FXI and FXIa, locking both in an inactive, zymogen-like conformation (97). In mouse and crab-eating macaque models, Abelacimab demonstrated good anticoagulant activity without bleeding events. The first-in-human study in healthy subjects demonstrated that a single subcutaneous dose of Abelacimab was safe and well-tolerated, with dose-and time-dependent prolongation of APTT and sustained suppression of functional FXI, effects lasting over 4 weeks (98). In a study evaluating the safety, pharmacokinetics, and pharmacodynamics of single intravenous and multiple subcutaneous doses of Abelacimab in healthy volunteers and AF patients, respectively a reduction in free FXI levels exceeding 99% was observed 1 hour after intravenous Abelacimab administration, with a drug half-life of 25 to 30 days. repeated Once-monthly subcutaneous administration can sustained reduction in free FXI concentrations and corresponding suppression of FXI-dependent coagulation activity. Abelacimab has good overall tolerability and no clinically significant bleeding events were observed, providing support for its subsequent clinical development (99). In a study involving 412 total knee arthroplasty patients receiving either a single intravenous dose of Abelacimab (30 mg, 75 mg, or 150 mg) or 40 mg enoxaparin subcutaneously postoperatively. The results showed that compared with the enoxaparin group, patients treated with Abelacimab had a lower incidence of VTE and a lower risk of bleeding (100). A trial investigating bleeding risk in patients with AF and moderate-to-high stroke risk receiving subcutaneous Abelacimab (150 mg or 90 mg monthly) or oral rivaroxaban (20 mg daily) was terminated early due to a greater-than-anticipated reduction in bleeding events with Abelacimab. Existing experimental data suggest that in patients with AF who are at moderate to high risk of stroke, treatment with Abelacimab significantly reduces free FXI levels and results in fewer bleeding events compared to therapy with rivaroxaban. The study demonstrated that Abelacimab, relative to rivaroxaban, effectively produced sustained reductions in free FXI concentrations and consistently reduced bleeding, irrespective of concomitant antiplatelet therapy. Notably, a greater absolute reduction in bleeding was observed in patients requiring APT. These findings propose that Abelacimab may serve as a safe anticoagulant option for AF patients necessitating combination therapy with APT (101, 102). A systematic study that included AZALEA-TIMI 71, OCEANIC-AF, and PACIFIC-AF indicates that FXI inhibitors significantly reduce the risk of bleeding and improve safety. However, compared with the control group, the antithrombotic efficacy of Abelacimab is still uncertain (103). Currently, two Phase III clinical trials are evaluating Abelascimab. One is a randomized, double-blind, placebo-controlled multicenter study comparing its effects on ischemic stroke or systemic embolism in patients with AF (NCT05712200, initiated in January 2023). The other is a randomized, open-label, blinded endpoint assessment multicenter study comparing its efficacy with dalteparin in patients with cancer-related venous thromboembolism in the gastrointestinal/urogenital system (NCT05171075, expected to be completed in February 2027).

Osocimab (Bay 1213790) is an anti-FXIa antibody generated via phage display technology. It binds to epitopes on FXIa, inducing structural rearrangement of the FXIa active site and thereby inhibiting FXIa activity (104). *In vitro* studies have demonstrated that Osocimab preserves platelet aggregation, prolongs clotting time, and reduces end fibrin content (105). A phase I study in healthy adults confirmed that intravenous Osocimab was safe and well tolerated, with no bleeding or anti-drug antibody formation, APTT prolongation and FXI coagulant activity were dose-dependent, and systemic exposure increased with dose (106). A phase II trial involving 813 patients who underwent knee joint replacement surgery in 54 hospitals from 13 countries compared the anticoagulant effects of Osocimab (administered by weight), enoxaparin (40 mg, subcutaneous, once daily, before surgery or after surgery), and apixaban (2.5 mg, orally, twice daily, after surgery). The results showed that administering 1.8 mg/kg of osusimab before surgery significantly reduced the risk of VTE 10–13 days after surgery, and was superior to enoxaparin. Its incidence of major bleeding or clinically relevant non-major bleeding was lower than that of enoxaparin but slightly higher than that of apixaban (107). A phase II randomized, double-blind, placebo-controlled trial involving 704 patients with renal failure undergoing hemodialysis evaluated the safety of Osocimab. The results showed that both the low-dose and high-dose Osocimab could reduce the incidence of clinically relevant bleeding compared to the placebo, but the incidence of the composite adverse event endpoint slightly increased (108).

Gruticibart (AB023), is a humanized anti-FXI antibody exhibiting 70% homology with the FXI antibody 14E11, both of which target the A2 domain of FXI. In murine models, 14E11 inhibits the activation of FXI by FXIIa, thereby exerting an antithrombotic effect while not affecting the activation of FXI by FXIa or thrombin, thus preserving hemostatic function (109). Gruticibart inhibits the activation of FXI by human FXIIa and suppresses the reverse activation of FXII by FXIa, without inhibiting the activation of FIX by FXIa or the activation of FXI by thrombin (110). In the initial Phase I clinical trial involving human subjects, Gruticibart did not result in increased bleeding or elevated prothrombin levels, and no serious adverse events were reported (111). A randomized, double-blind phase II trial enrolled 24 patients with ESRD who were undergoing chronic hemodialysis. The results showed that a single dose of Gruticibart(0.25 or 0.5 mg/kg) was well tolerated and could reduce the formation of new blood clots within the dialyzer (112). A prospective single-arm study enrolled cancer patients who underwent central venous catheter insertion and received a single dose of Gruticibart (2 mg/kg) followed by follow-up. The results showed that the incidence of catheter-related thrombosis (CAT) was 12.5% (40.0% in the control group), and the APTT was significantly prolonged compared to the baseline. No infusion reaction, drug-related adverse events, or clinically relevant bleeding was observed (113).

SHR-2004 is a humanized monoclonal antibody that selectively binds to FXI and FXIa. Phase I studies demonstrated that a single intravenous or subcutaneous dose of SHR-2004 (0.1–4.5 mg/kg) was well tolerated, with plasma exposure increasing in a dose-dependent manner and a geometric mean half-life of 11.6–13.0 days. Both FXI coagulant activity inhibition and APTT prolongation were dose- and time-dependent; after the highest intravenous dose, FXI coagulant activity was nearly completely inhibited and mean APTT extended to nearly threefold baseline (114). The phase III trial of SHR-2004 (NCT06825416) enrolled 1,166 patients undergoing elective unilateral total knee arthroplasty and employed a multicenter, randomized, quadruple-blind, active-controlled design. Results showed that a single intravenous dose of SHR-2004–90 mg administered 4–8 hours postoperatively reduced the incidence of VTE by 51% compared with enoxaparin, with a comparable safety profile. This “single-dose, full-course coverage” regimen offers significant clinical convenience over daily injectable anticoagulants (115). Currently, the clinical studies of SHR-2004 include: a phase I randomized double-blind, placebo-controlled dose-escalation trial (NCT05369767), which assesses safety, tolerability, pharmacokinetics and pharmacodynamics in healthy subjects. An open-label phase Ib trial (NCT06280768), which evaluates the safety and tolerability of multiple subcutaneous injections in patients with AF, with the secondary endpoints being pharmacokinetics, pharmacodynamics and immunogenicity. And another multicenter, randomized, controlled open-label phase II trial (NCT06220123) has been completed, aiming to evaluate the efficacy and safety of SHR-2004 in preventing VTE after ovarian cancer surgery, but the results have not been published yet.

Bay 1831865 is a humanized monoclonal antibody that selectively binds to FXI and FXIa. In healthy volunteers receiving single intravenous doses ranging from 3.5 to 150 mg or single subcutaneous dose of 150 mg, intravenous administration showed a rapid onset of action with dose-dependent APTT prolongation and enhanced suppression of FXI coagulant activity at higher doses. Subcutaneous administration produced similar effects but with a slower onset. Bay 1831865 was well tolerated throughout the trial, with no evidence of increased bleeding risk (116).

SKB336 is an innovative humanized monoclonal antibody that exerts its effect through high - affinity binding to FXI and FXIa, rather than directly inhibiting FXIa enzyme activity or binding to other coagulation factors. In a phase I dose-escalation study (0.1–4 mg/kg), all dose groups showed good tolerability, with no bleeding events, deaths, or serious adverse events; no dose-related trend in adverse event incidence was observed. Pharmacokinetically, Tmax and AUC increased with dose, with a mean elimination half-life of 21.3–33.5 days. Pharmacodynamically, APTT was prolonged in a dose-dependent manner, and sustained FXI coagulant activity inhibition was maintained (117).

BJTJ-1837, a humanized antibody, can bind to the protease domain of FXI and FXIa with high affinity. BJTJ - 1837 completely inhibited the activation of FXI induced by activated FXII and thrombin. BJTJ - 1837 also showed potent anticoagulant activity in human and cynomolgus monkey plasma, as measured by APTT. In addition, BJTJ - 1837 showed promising antithrombotic activity in a cynomolgus monkey model of arterio - venous shunt thrombosis, with dose - dependent protection and without bleeding adverse effects (118).

## Suppression at the source: gene silencing strategies with ASOs and siRNA

ISIS 404071 is the first generation of FXI -ASO, which binds to FXI mRNA through specific hydrogen bonds or hydrophobic interactions. Preliminary studies in mouse models have shown that subcutaneous injection of ISIS 404071 for three weeks can specifically reduce the FXI mRNA level in the liver and the FXI protein level in the plasma, accompanied by an increase in the APTT, and without affecting PT. Further observations using *in vivo* microscopes revealed that ISIS 404071 can significantly inhibit platelet aggregation and thrombosis at the site of vascular injury (119). Studies in mouse models have demonstrated that FXI-ASO combined with enoxaparin exhibits significantly greater antithrombotic activity compared to the small - molecule inhibitors of FXa or enoxaparin alone, without increasing the bleeding risk (120).

ISIS-FXIRX (IONIS-FXI-Rx), a second-generation FXI-ASO, was administered to macaques at 25 mg/kg twice weekly for 13 weeks. Results demonstrated good tolerability of ISIS-FXIRX, with significant reductions in hepatic FXI RNA and plasma FXI protein and activity levels. The study also observed increased APTT in the treatment group, though no effects were noted on PT or platelet function (121). Furthermore, multiple animal models have validated the aforementioned results (122, 123). These findings suggest that antisense drug technology may represent an attractive approach for specifically targeting FXI in the treatment of thrombotic disorders. In a study involving 300 patients undergoing knee replacement surgery, the results indicated that patients receiving the 300 mg dose of ISIS-FXIRX demonstrated superior VTE prevention compared to enoxaparin, while the 200 mg FXI - ASO showed no significant difference in efficacy versus enoxaparin. Regarding bleeding, the incidence was lower in both FXI - ASO groups compared to the enoxaparin group (10). In a two - part, random, double - blind, placebo - controlled Phase II trial, patients with ESRD undergoing hemodialysis received either 200 mg or 300 mg of ISIS - FXIRX or placebo for 12 weeks. Results demonstrated that ISIS-FXIRX reduced FXI coagulant activity in ESRD patients undergoing hemodialysis without drug - related adverse events (124).

Fesomersen (BAY2976217 or FXI-LICA or ION-957943) is a second-generation, tri-arm N-acetylgalactosamine (GalNAc)-conjugated 2’-MOE chimeric ASO inhibitor of FXI, sharing the same RNA sequence as ISIS-FXIRX. In a double-blind, placebo-controlled, dose-escalation Phase I study, the exposure demonstrated a dose-dependent increase within the 10–120 mg range. Moreover, FXI coagulant activity exhibited a significant reduction in a dose-dependent manner across all dose groups (125). A Phase I study of subcutaneous injection of Fesomersen in healthy volunteers demonstrated that Fesomersen was safe and well - tolerated. There were no severe bleeding events. There was rapid absorption following a single subcutaneous dose, followed by a bimodal concentration decline. There was a dose - dependent reduction in FXI coagulant activity and prolongation of APTT (126). Additionally, a multiple - dose Phase II, randomized, double - blind, placebo - controlled study of Fesomersen aimed to further evaluate its safety, tolerability, and pharmacokinetics (absorption, distribution, and excretion) in ESRD patients requiring hemodialysis. Results demonstrated a dose - dependent reduction in FXI levels with Fesomersen with no significant difference in bleeding rates (127).

RBD4059 is a siRNA conjugated with a liver - targeting GalNAc ligand, which regulates mRNA translation to inhibit FXI synthesis by enhancing ASGPR - mediated endocytosis - mediated uptake. Pharmacokinetics/Pharmacodynamics studies in mice and cynomolgus monkeys demonstrated rapid absorption of RBD4059. Peak plasma concentrations were reached in approximately 1.6 hours, and it had a short half - life, enabling rapid hepatic distribution. A single 9 mg/kg dose of RBD4059 achieved a maximum inhibition of 75.5% for FXI mRNA in the liver and a maximum inhibition rate of 76.5% for FXI coagulant activity, along with a significant prolongation of APTT. RBD4059 demonstrated favorable antithrombotic effects. It showed no significant difference in efficacy compared to enoxaparin 4 mg/kg, while not increasing the bleeding risk (128). A randomized, single-blind, placebo-controlled Phase I study is currently underway to evaluate the safety, tolerability, pharmacokinetics, and pharmacodynamics of single ascending doses of subcutaneously administered RBD4059 in healthy subjects (NCT05653037). A random, double-blind, placebo-controlled Phase IIa trial is currently underway, initiated in August 2024, to evaluate the safety, pharmacokinetics and pharmacodynamics of repeated subcutaneous administration of RBD4059 in participants with stable coronary artery disease (NCT06717074).

SRSD107 is a chemically modified double - stranded siRNA specifically targeting human FXI mRNA. A Phase I randomized double - blind placebo - controlled single - dose escalation study involving 40 healthy Australian adults demonstrated that SRSD107 was safe and well - tolerated, with moderate absorption and rapid elimination. FXI coagulant activity remained suppressed for at least 12 weeks post - administration, accompanied by significant prolongation of APTT and no notable changes in PT (129).

## Other innovative strategies: peptide-based, protein-based and synthetic compound inhibitors

Shp4 is a Kunitz domain-containing protein secreted by schistosomes, exhibiting a Ki value of 3.35 nanometer and inhibiting FXIa in a non-heparin-dependent manner. In a FeCl₃-induced carotid thrombosis model, Shp4 was found to specifically inhibit FXIa activity, with negligible effects on FXIIa or thrombin, and it only mildly suppresses plasmin activity. Shp4 inhibits the APTT pathway without significantly affecting the extrinsic or common coagulation pathways (130).

Protease nexin-2 (PN2) is a Kunitz-type inhibitor (KPI) secreted from platelet α-granules into plasma, where it inhibits FXIa activity with approximately 775-fold greater potency than trypsin (131). In an investigation examining the effects on thrombosis in mouse carotid and middle cerebral arteries, intravenous administration of PN2KPI prolonged the clotting times in both human and mouse plasma, inhibited FXIa activity in both plasma samples, and reduced carotid thrombosis in animal models (132).

rFasxiatorN17R, L19E, based on the Kunitz-type protease inhibitor isolated and engineered from the venom of the banded krait (Bungarus fasciatus), achieved antithrombotic efficacy comparable to that of a therapeutic dose of unfractionated heparin (UFH) in a FeCl_3_-induced rat carotid artery thrombosis model at an intravenous dose of 2mg/kg. Crucially, the tail bleeding time it induced was reduced by more than threefold compared to the UFH group, highlighting its superior hemostatic safety profile. In a mouse inferior vena cava ligation model of venous thrombosis, a 10 mg/kg subcutaneous dose of rFasxiatorN17R,L19E showed similar efficacy to enoxaparin in reducing thrombus weight, while causing a comparable level of bleeding (133).

DX-88mut is a serine protease synthesized using Loop1 (TGPCRAMISR) and Loop2 (FYGGC) from protease linker protein-2, which is capable of selectively inhibiting FXIa. At 1 μmol/kg, DX-88mut significantly prolonged APTT in mouse, rat, and human plasma and effectively inhibited FeCl_3_-induced carotid thrombosis in mice. Furthermore, DX-88mut demonstrated no apparent bleeding risk at 5 μmol/kg (134).

The compound (S)-10h (containing a triazole benzoic acid fragment) is a potent FXIa inhibitor with inhibitory activity approximately 3 times that of Asundexian, exhibiting 150-fold higher selectivity for PKa compared to Asundexian (13-fold vs. 150-fold) and 100,000-fold higher selectivity for FXa and thrombin (5,000-fold vs. 100,000-fold), demonstrating potent anticoagulant activity (135). FXIa inhibitors based on the 1-(pyridin-2-ylmethyl)pyridine-2(1H)-ketone structure (e.g., 37, 39b, 43, 46b) exhibit high selectivity and potent inhibitory effects, with VTE efficacy comparable to or superior to Asundexian. Compound 43 significantly reduced thrombosis incidence in FeCl_3_-induced arterial thrombosis models in mice and rabbits, demonstrating superior efficacy to Asundexian without increasing bleeding risk, and demonstrated good safety profiles in murine studies (136).

Compound FE12, a synthetic lead compound, demonstrates high selectivity in inhibiting FXIa activity. *In vitro*, FE12 selectively prolongs APTT in a dose-dependent manner, without affecting PT, confirming its specific action on the intrinsic coagulation pathway. In a FeCl₃-induced rat carotid artery thrombosis model, FE12 (2 mg/kg, intravenous injection) effectively inhibited thrombus formation to an extent comparable to that of Asundexian. More notably, in a mouse tail transection bleeding assay, FE12 did not cause a statistically significant prolongation of bleeding time, indicating a minimal impact on hemostatic function and a potentially superior safety window regarding bleeding risk. Its development exemplifies a successful structure-based optimization, providing a foundation for the development of safer and more selective next-generation anticoagulant drugs (137).

## Mechanistic differences between the major therapeutic platforms

Although all FXI/FXIa-targeted treatment regimens are designed to inhibit FXI-dependent coagulation, different platforms vary considerably in mechanism of action, pharmacological properties, pharmacokinetics/pharmacodynamics characteristics, and clinical applicability. This leads to heterogeneous therapeutic effects across different diseases. The differences in efficacy, however, are not determined solely by the drug itself; they also depend on two key factors: the FXI/FXIa inhibition pattern enabled by the platform, and the degree to which a specific thrombotic disease relies on FXI-mediated coagulation amplification. FXI/FXIa is mainly involved in the amplification phase of thrombin generation, rather than the initial TF-triggered hemostasis. Accordingly, the optimal therapeutic window for FXI/FXIa inhibition lies in clinical settings where pathological thrombosis heavily depends on FXI-mediated coagulation amplification, while physiological hemostasis is mainly compensated and maintained via other pathways. This framework helps explain why FXI inhibitors show consistent efficacy for preventing postoperative thrombosis in orthopedic surgery, but display heterogeneous effects in chronic cardioembolic thrombotic diseases such as AF (52, 138, 139).

Oral small-molecule FXIa inhibitors reversibly bind to the catalytic site of FXIa to inhibit its enzymatic activity, without altering plasma FXI protein levels. Their pharmacodynamic effects are exposure-dependent and weaken as plasma drug concentrations drop. These agents have a rapid onset of action, are convenient for oral administration, and feature reversible binding properties, making them potentially suitable for clinical settings that require rapid adjustment of anticoagulation intensity, such as ACS and secondary prevention after ischemic stroke (72, 73, 140). Nevertheless, the two agents differ in metabolic pathways and renal clearance: Milvexian is a substrate of CYP3A4/5 and P-glycoprotein and is primarily metabolized by CYP3A4, while Asundexian is mainly metabolized by carboxylic esterase (141, 142). The inhibitory effect of FXIa inhibitors is concentration-dependent, which means reduced drug exposure, drug interactions, or missed doses can all result in insufficient targeted inhibition. Although therapeutic efficacy can be evaluated via FXIa activity, drug concentration, and APTT, APTT measurement relies on specific reagents and non-specific quantitative indicators (143). Routine monitoring is generally not performed in clinical practice, but such monitoring may still have certain practical clinical value under special circumstances, such as when a bleeding event occurs or before emergency surgery. Accordingly, these pharmacokinetic differences may lead to clinically significant efficacy variations in conditions requiring continuous anticoagulation, such as AF.

Monoclonal antibodies target specific epitopes of FXI or FXIa, exerting their effects by preventing FXI activation or directly neutralizing FXIa (97, 104). Compared with oral small-molecule FXIa inhibitors, they feature higher specificity, longer target occupancy, and a longer duration of efficacy. These properties mainly depend on the antibody clearance rate and target turnover rate, rather than the CYP-mediated liver metabolism process (144, 145). However, their long-acting effect slows the rapid recovery of hemostatic function during severe bleeding or emergency surgery, and perioperative management as well as reversal strategies still require further investigation. Even within the same monoclonal antibody platform, clinical outcomes differ due to variations in target form, binding epitope, mechanism of action, dosing regimen, and administration timing (146). For example, in TKA, Osocimab showed dose- and timing-dependent thromboprophylactic efficacy: postoperative doses of 0.6–1.8 mg/kg were noninferior to enoxaparin, while a preoperative dose of 1.8 mg/kg was superior to enoxaparin; and the 0.3 mg/kg regimen failed to meet the prespecified noninferiority criterion (107). Similarly, a single postoperative intravenous dose of Abelacimab provided dose-dependent protection against VTE (100). These findings indicate that clinical efficacy not only depends on the treatment platform itself, but may also be associated with epitope specificity, the degree and duration of FXI/FXIa inhibition, dose selection, and administration timing.

Unlike direct FXI inhibitors, ASO- and siRNA-based approaches work by reducing hepatic FXI synthesis, which in turn lowers circulating FXI levels. Specifically, siRNA therapies trigger FXI mRNA degradation via the RISC, while ASOs promote FXI mRNA breakdown through RNase H1-mediated mechanisms (147).

Given that their pharmacodynamic effects rely on the depletion of hepatic FXI mRNA and the subsequent turnover of the pre-existing circulating FXI protein pool, these agents generally have a slower onset of action than direct FXI inhibitors, but can achieve sustained suppression of FXI synthesis and plasma FXI coagulant activity (148). Their pharmacodynamic effects can be evaluated by measuring FXI antigen levels and FXI coagulant activity, and APTT can be used as an indirect functional marker (10).

Given that their pharmacodynamic effects rely on the depletion of hepatic FXI mRNA and the subsequent turnover of the pre-existing circulating FXI protein pool, these agents typically have a slower onset of action than direct FXI inhibitors, yet they are able to achieve sustained suppression of FXI synthesis and plasma FXI coagulant activity. For example, FXI-ASO therapy has been shown to produce sustained reductions in FXI coagulant activity in hemodialysis patients, providing pharmacodynamic proof of concept and supporting the biological rationale for FXI/FXIa-targeted therapy in patients with ESKD (124, 127). Because pharmacodynamics recovery relies on the synthesis and accumulation of new FXI protein, the prolonged effects of these agents may present challenges for urgent drug reversal and perioperative management (146).

## Disease biology affects the clinical performance of FXI inhibitors

Although arterial and venous thrombi both share downstream processes including thrombin generation and fibrin formation, they differ fundamentally in triggering factors, hemodynamic environments, cellular composition, and the degree of dependence on FXI-mediated coagulation amplification. These differences are likely to directly influence how much benefit FXI inhibitors can deliver for different types of thrombotic diseases. VTE often occurs in areas with slow blood flow or stasis. Unlike most types of arterial thrombosis, VTE usually does not involve obvious vascular wall damage, and is closely related to the Virchow triad (blood stasis, endothelial activation/function impairment, hypercoagulable state), as well as systemic or local hypercoagulable states (149).Venous stasis leads to local hypoxia and endothelial activation, upregulates P-selectin, recruits leukocytes and extracellular vesicles carrying TF, and initiates coagulation through the TF-VIIa pathway; at the same time, NETs and other factors can activate the contact pathway, promoting FXII and subsequent FXI activation (150–154). Initial thrombin further activates FXI, forming a positive feedback amplification loop, enhancing FIX/FX activation, triggering thrombin burst and fibrin deposition. Therefore, FXI/FXIa mainly acts as an amplification factor rather than an initiating factor in VTE (138). This might explain why the efficacy of VTE prevention in orthopedic surgeries (such as TKA) is stable - this state combines tissue damage, postoperative stasis, endothelial disorder and inflammation, forming a highly FXI-dependent thrombus microenvironment (155). Multiple studies (FXI-ASO, Milvexian, Abelacimab) have shown that they are not inferior to enoxaparin in reducing the incidence of postoperative VTE, and the increase in bleeding is limited, supporting the high dependence of this type of thrombus on FXI/FXIa (10, 90, 100). However, whether these findings can be extrapolated to all forms of VTE requires further validation in additional clinical trials.

Unlike venous thrombosis, atherothrombotic arterial events generally occur under high-shear conditions, secondary to atherosclerotic plaque rupture, plaque erosion, or endothelial injury. Plaque rupture exposes thrombogenic extracellular matrix components, and in particular, exposes TF, which triggers platelet activation and coagulation simultaneously. Under the high-shear arterial environment, the interaction between vWF and platelet glycoprotein Ibα (GPIbα) mediates the initial capture and rolling of platelets; subsequently, collagen promotes stable platelet adhesion and activation through glycoprotein VI (GPVI) and integrin α_2_β_1_, triggering the release of agonists such as ADP and thromboxane A₂, and inducing conformational activation of integrin αIIbβ3 (GPIIb/IIIa). Activated αIIbβ3 binds fibrinogen and vWF to connect adjacent platelets, promoting platelet aggregation. At the same time, TF exposed inside the plaque binds to coagulation factor VII/VIIa to initiate the extrinsic coagulation pathway and activate FIX and FX, which in turn generate thrombin. Thrombin further activates platelets and converts fibrinogen to fibrin, promoting thrombus growth and stabilization (156–159). Thus, platelet activation is the dominant initiating mechanism of atherothrombotic arterial events. Accordingly, antiplatelet therapy remains the cornerstone of treatment for ACS and non-cardioembolic ischemic stroke (160–162). In these conditions, FXI/FXIa inhibition is not intended to replace antiplatelet therapy, but rather to suppress residual coagulation-driven thrombus propagation after platelet-mediated thrombus initiation. The therapeutic rationale is to attenuate residual coagulation-driven thrombin generation, fibrin formation, and thrombus extension after the platelet-mediated initiation phase, with the goal of reducing recurrent ischemic events without significantly impairing primary hemostasis. This distinction is critical for interpreting clinical trial outcomes. In atherothrombotic arterial disease, FXI inhibitors are mainly studied as an adjunctive strategy added to standard antiplatelet therapy, designed to suppress coagulation-driven thrombin generation and fibrin formation while preserving primary hemostasis. In contrast, for AF-associated thromboembolism, FXI/FXIa inhibition has been evaluated as a potential replacement for conventional anticoagulants, representing a fundamentally different biological and therapeutic context.

AF-associated intracardiac thrombi most commonly originate from the left atrial appendage (LAA). The loss of effective atrial contraction leads to severe blood stasis within the LAA, which is further exacerbated by its narrow, blind-ended anatomical structure. This structure promotes local flow disturbance and creates low-shear conditions that are particularly favorable for thrombus formation (163, 164). Although AF-related thromboembolism clinically presents as arterial embolic events, the underlying thrombi are typically fibrin-rich or mixed thrombi, rather than the platelet-dominant thrombi that characterize atherosclerotic arterial thrombosis (165). The thrombotic mechanism of AF shares some similarities with postoperative VTE: thrombin generation and subsequent FXI activation both contribute to thrombin amplification and fibrin formation. However, AF differs fundamentally from postoperative VTE in its biological context. Postoperative VTE develops in the setting of acute tissue injury, inflammation, and transient activation of coagulation pathways, while AF-associated thrombosis occurs under chronic conditions characterized by persistent blood stasis and endothelial dysfunction within the LAA. In this environment, sustained tissue factor expression and continuous activation of the extrinsic coagulation pathway may drive thrombin generation through mechanisms that are not fully dependent on FXI-mediated coagulation amplification. This biological distinction may help explain the divergent outcomes observed in the OCEANIC-AF and OCEANIC-STROKE trials. In OCEANIC-AF, Asundexian showed a favorable bleeding profile but failed to meet noninferiority compared with apixaban for preventing stroke and systemic embolism, indicating that FXIa inhibition may be insufficient as a replacement for conventional anticoagulation in AF (69). In contrast, in OCEANIC-STROKE, FXIa inhibition was evaluated as an adjunctive therapy added to antiplatelet treatment, designed to suppress residual coagulation amplification after platelet-mediated thrombus initiation — an indication that aligns more closely with the biological role of FXI (68).

Collectively, these observations indicate that the clinical benefit of FXI/FXIa inhibition is determined not only by the pharmacological characteristics of the inhibitor itself but also by the degree of FXI dependency within specific thrombotic phenotypes. VTE represents a thrombotic condition with relatively high dependence on FXI-mediated coagulation amplification, whereas atherothrombosis generally requires combined targeting of platelet activation and coagulation amplification. In contrast, AF-associated cardioembolism represents a distinct thrombotic phenotype in which the quantitative contribution of FXI-mediated coagulation amplification remains incompletely defined.

Chronic thrombotic disorders associated with an elevated risk of bleeding, including ESKD and CAT, represent potentially attractive indications for FXI/FXIa-targeted therapy. Both conditions are characterized by persistent inflammatory and procoagulant activation, whereas conventional anticoagulation is accompanied by a substantial risk of bleeding (146, 166). In ESKD, thrombosis arises from complex interactions among endothelial dysfunction, chronic inflammation, tissue factor expression, platelet activation, and repeated activation of contact pathways during hemodialysis. Exposure of blood to artificial dialysis membranes activates FXII–dependent pathways, thereby promoting FXI activation and thrombin amplification. Accordingly, FXI may be involved in the interconnection among extracorporeal contact activation, dialysis-circuit clotting, vascular-access thrombosis, and systemic coagulation activation (146). Phase II studies provide preliminary clinical support for this rationale. In patients with ESKD receiving hemodialysis, IONIS-FXIRx produced dose-dependent reductions in FXI coagulant activity and was associated with less visible clot formation within the dialysis circuit, although the study was not powered to determine effects on major thrombotic outcomes (124). Similarly, once-monthly Fesomersen produced sustained, dose-dependent reductions in FXI levels, while lower predicted FXI levels were associated with reduced dialysis-circuit clotting and arteriovenous-access thrombosis (127). These results demonstrate pharmacodynamic activity and provide proof of mechanism, adequately powered phase III trials are required to establish effects on clinically important thrombosis, bleeding, and mortality. Similarly, CAT represents another chronic inflammatory and prothrombotic state in which FXI/FXIa inhibition may offer therapeutic advantages. Tumor cells promote coagulation through tissue factor expression, inflammatory cytokine release, platelet activation, and NETosis, resulting in sustained thrombin generation and fibrin deposition. Because cancer patients frequently require prolonged thromboprophylaxis while simultaneously facing an elevated risk of bleeding, FXI/FXIa inhibition may provide an attractive strategy by selectively targeting pathological coagulation while preserving hemostatic capacity. However, clinical evidence in CAT remains limited, and future studies are required to determine whether FXI dependency varies according to tumor type, disease stage, and treatment context (166, 167).

Overall, the clinical performance of FXI inhibitors is determined by the interaction between the pharmacological properties of the therapeutic platform and disease-specific thrombotic biology. Short-acting oral FXIa inhibitors may be particularly suitable for acute clinical settings requiring rapid and reversible modulation, such as ACS and secondary prevention of ischemic stroke. Long-acting antibodies, ASO-, and siRNA-based strategies may be more appropriate for chronic conditions requiring sustained FXI suppression, including ESKD and potentially CAT. The contrasting outcomes observed in TKA, OCEANIC-STROKE, and OCEANIC-AF highlight that FXI/FXIa inhibition should not be considered a universal replacement strategy for conventional anticoagulation. Instead, the future success of FXI/FXIa-targeted therapies will depend on identifying thrombotic phenotypes in which FXI-mediated coagulation amplification constitutes a dominant and therapeutically accessible pathway.

## Discussion

Traditional anticoagulant drugs can effectively inhibit thrombosis, such as direct FXa inhibitors or thrombin inhibitors, but their effects lack selectivity and often damage normal hemostasis functions, resulting in a significant increase in bleeding risk (27). Moreover, these drugs have application contraindications or lack safety evidence in special populations such as those with ESRD or heparin-induced thrombocytopenia. FXI/FXIa plays a crucial amplification role in pathological thrombosis, but its contribution to physiological hemostasis is relatively limited. This unique biological characteristic provides a theoretical basis for targeting FXI/FXIa - aiming to achieve the separation of antithrombotic effects and the preservation of hemostatic functions, thereby overcoming the inherent limitations of traditional anticoagulant therapy.

FXI inhibitors exhibit promising efficacy across several thrombotic settings. These agents effectively prevent VTE following TKA (10, 90, 100, 107), when used as adjunctive therapy to antiplatelet treatment, may reduce recurrent ischemic events in patients with AMI or non-cardioembolic ischemic stroke (168). Regarding safety, FXI inhibitors have consistently demonstrated lower bleeding risks compared with enoxaparin or DOACs across different clinical indications (169). For patients with ESRD undergoing hemodialysis - a group with extremely limited safe anticoagulation options - both IONIS-FXIRx and Fesomersen show dose-dependent FXI inhibition effects and do not significantly increase the incidence of bleeding events (124, 127).

However, the clinical efficacy of FXI/FXIa inhibition is not universal but depends on the degree to which a specific thrombus phenotype relies on the FXI-mediated coagulation amplification pathway. In postoperative VTE after major orthopedic surgeries (such as TKA), the postoperative tissue damage, venous stasis, and inflammatory response jointly create a procoagulant microenvironment that is highly dependent on the FXI amplification effect. Multiple FXI/FXIa-targeting strategies using different mechanisms (ASOs, small molecule inhibitors, monoclonal antibodies) have shown the ability to effectively prevent postoperative VTE, with a relatively low incidence of clinically relevant bleeding events, suggesting that this phenotype is highly sensitive to FXI/FXIa inhibition (10, 90, 100, 107). On the contrary, atherosclerotic thrombosis begins with platelet activation as the initiating event, and the coagulation pathway mainly participates in the stabilization and expansion of the thrombus (156–159). Therefore, FXI/FXIa inhibition cannot replace antiplatelet therapy, but it can be used as an auxiliary strategy to weaken the residual thrombus expansion driven by coagulation after platelet activation. The Phase II PACIFIC-Stroke and Phase III OCEANIC-Stroke trials demonstrated that adding Asundexian to antiplatelet therapy significantly reduced recurrent ischemic events in patients with non-cardioembolic ischemic stroke, without increasing major bleeding. This was the first time at the Phase III level to verify the clinical value of the “antiplatelet combined with FXI/FXIa inhibition” dual-pathway strategy (68).

For AF-related cardiogenic embolism, it presents a unique intermediate phenotype: although thrombosis occurs in a low-flow environment and is rich in fibrin, its background is the continuous expression of tissue factor and chronic activation of the exogenous coagulation pathway, and it may not completely rely on FXI amplification (163, 164). In the Phase III OCEANIC-AF trial, although Asundexian had good bleeding safety, it failed to achieve non-inferiority compared to apixaban, suggesting that selective FXI/FXIa inhibition is not sufficient to replace direct FXa inhibitors in typical AF populations (68). For chronic thrombotic diseases with high bleeding risks such as ESKD and CAT, FXI/FXIa inhibition has potential advantages - in the case of end-stage renal disease, the contact pathway is repeatedly activated during hemodialysis, and in the case of cancer-related thrombosis, the tumor-related inflammation continuously drives coagulation. Early clinical trials showed that FXI-ASO or Fesomersen could reduce thrombus formation in dialysis circuits and had a bleeding risk comparable to placebo, but the clinical hard endpoint evidence awaits confirmation in Phase III studies.

Different pharmacological platforms confer differentiated clinical applicability to FXI inhibitors. Oral small molecule drugs (such as Asundexian, Milvexian) have rapid onset and reversible effects, making them suitable for scenarios requiring rapid intervention, such ACS or secondary prevention of ischemic stroke; monoclonal antibodies and GalNAc-conjugated ASOs or siRNAs can achieve sustained and stable FXI inhibition, supporting low-frequency administration and being more suitable for chronic long-term prevention, but their long-lasting characteristics also bring challenges in perioperative management and emergency reversal. Currently, there is no validated specific reversal strategy, and the potential long-term effects of sustained FXI/FXIa inhibition on immune homeostasis, infection defense, etc. are still unclear. Systematic safety follow-up is urgently needed. In addition, FXI is involved in complement regulation, NETosis, and inflammatory signaling pathways, suggesting its possible role in immune thrombosis (such as sepsis, COVID-19-related thrombosis). However, this field is still in the exploration stage, and further mechanisms and clinical studies are needed for verification.

Notably, substantial progress has been made in the chemical modification of FXI inhibitors. GalNAc-conjugated FXI-ASOs enhance the efficacy of FXI-ASO drugs, while specific modifications of the oligonucleotide-deoxynucleotide gap region in toxic phosphorothioate (PS) gapmer ASOs improve both safety and activity (170, 171). Chemically modified FXI inhibitors, such as RBD4059 and SRSD107, have entered clinical trials. These agents achieve rapid hepatic distribution and maintain FXI inhibition for several weeks to months following a single subcutaneous administration (128, 129). Such delivery platforms enable low-frequency dosing with predictable efficacy, offering the potential to significantly improve medication adherence in patients with chronic thrombotic diseases.

## Outstanding questions

Despite substantial advances in the development of FXI/FXIa-targeted therapies, several critical questions remain unresolved and require further investigation. In terms of laboratory monitoring, APTT is currently the most commonly used surrogate indicator for FXI coagulant activity. However, APTT is susceptible to interference from lupus anticoagulants, contact pathway activation, and variations in detection protocols, while also lacking standardized testing methods, making it difficult to precisely guide individualized drug administration. Developing standardized detection methods that can directly measure FXI antigen, FXI coagulant activity, or the drug-specific pharmacodynamic effects will be crucial for optimizing dosing regimens, supporting perioperative clinical decision-making, and improving treatment safety.

Furthermore, although FXI inhibitors have shown favorable safety profiles and promising antithrombotic effects across multiple thrombotic conditions, it remains unclear which patient populations will derive the greatest clinical benefit from these agents. To date, no clinical trials have evaluated FXI inhibitors for the treatment of established DVT, and evidence supporting their efficacy in arterial thrombotic diseases remains incomplete. Future studies should focus on identifying biomarkers that can predict FXI-dependent thrombotic phenotypes and establishing patient stratification strategies. Such work may facilitate the transition from a one-size-fits-all anticoagulation model to precision anticoagulation, where treatment selection is guided by thrombotic mechanism, FXI coagulant activity, inflammatory status, and individual bleeding risk. In addition, the optimal clinical positioning of FXI inhibitors across different patient populations has yet to be clarified, and patients with high bleeding risk, chronic kidney disease, or contraindications to conventional anticoagulants are considered potential target populations.

Moreover, the development of long-acting FXI/FXIa-targeting delivery platforms, including monoclonal antibodies, ASOs, and siRNA-based therapies, is an important research direction in this field. These approaches achieve prolonged suppression of FXI coagulant activity, reduce dosing frequency, and may improve medication adherence among patients requiring long-term thromboprophylaxis. However, their sustained pharmacological effects also bring new safety challenges. Unlike short-acting small-molecule FXIa inhibitors, the anticoagulant effects of long-acting FXI/FXIa inhibitors cannot be rapidly reversed in cases of major bleeding, emergency surgery, or severe trauma, and no clinically validated specific reversal strategy is currently available for these agents. Therefore, developing FXI-specific antidotes, reversible FXI/FXIa inhibition platforms, or strategies that can rapidly restore FXI function remains an important priority for future research.

In addition, the long-term consequences of sustained FXI/FXIa suppression are still not fully understood. Given the newly identified role of FXI/FXIa in immunothrombosis, prolonged inhibition may potentially affect immune homeostasis, pathogen clearance, infection susceptibility, and inflammatory regulation. The prolonged duration of action also creates additional challenges for perioperative management and emergency reversal. Therefore, future FXI/FXIa-targeted therapeutic strategies should not blindly pursue maximal FXI/FXIa suppression; instead, the most appropriate platform should be selected according to the specific characteristics of each disease and patient. Long-term clinical studies are needed to confirm whether sustained FXI/FXIa suppression can maintain an acceptable balance among thrombotic protection, bleeding risk, and immune function.

## Future perspectives

Several ongoing phase III clinical trials are expected to further clarify the clinical positioning of FXI inhibitors, and may reshape future anticoagulation strategies across different thrombotic phenotypes.

In AF, the LIBREXIA-AF trial is evaluating whether a higher-intensity, more frequent dosing regimen of the small-molecule FXIa inhibitor Milvexian can overcome the insufficient efficacy observed with Asundexian in the OCEANIC-AF trial. If Milvexian demonstrates non-inferiority relative to standard anticoagulation, FXIa inhibitors will regain potential as an alternative strategy for stroke prevention in AF. Conversely, if it fails to prove adequate efficacy, this will further support the view that selective FXIa inhibition may not be sufficient to replace DOACs in patients with typical high-risk AF driven by persistent coagulation activation. The LILAC-TIMI 76 trial adopts another strategic approach by focusing on AF patients with high bleeding risk, a population with limited available therapeutic options. If Abelacimab demonstrates effective prevention of thromboembolic events without causing excessive bleeding, it will establish FXI/FXIa inhibition as a clinically valuable option for carefully selected AF subgroups, and expand the application of FXI/FXIa-targeted therapies beyond the scope of conventional anticoagulation pathways.

In atherosclerotic thrombotic diseases, the LIBREXIA-STROKE trial (NCT05702034) is evaluating the efficacy and safety of Milvexian for preventing recurrent ischemic stroke in patients with a history of acute ischemic stroke or high-risk transient ischemic attack who are receiving background antiplatelet therapy. Together with results from the OCEANIC-STROKE trial, these findings will help determine whether FXIa inhibition can be established as an effective adjunctive strategy to antiplatelet therapy for secondary prevention of atherosclerotic thrombotic events.

Overall, the outcomes of these ongoing phase III trials will determine whether FXI inhibitors will ultimately serve as replacements for conventional anticoagulants, adjunctive therapies combined with antiplatelet agents, or specialized treatment options for populations with unmet clinical needs. More importantly, these studies will consolidate a core principle that has emerged from current FXI/FXIa research: the success of FXI/FXIa-targeted therapy depends not only on pharmacological potency, but also on the precise matching between drug properties and the specific thrombotic biology of the disease. Therefore, future anticoagulation strategies are likely to shift from a generalized anticoagulant approach toward precision-guided therapy tailored to thrombotic phenotype, FXI dependency, and individual risk profiles. Through this paradigm shift, FXI inhibitors may contribute to a new generation of anticoagulant therapies that achieve effective thrombosis prevention while preserving physiological hemostasis.

## References

[1] Wendelboe AM Raskob GE . Global burden of thrombosis. Circ Res. (2016) 118:1340–7. doi: 10.1161/circresaha.115.306841 27126645

[2] GBD 2021 Diseases and Injuries Collaborators . Global incidence, prevalence, years lived with disability (YLDs), disability-adjusted life-years (DALYs), and healthy life expectancy (HALE) for 371 diseases and injuries in 204 countries and territories and 811 subnational locations, 1990–2021: a systematic analysis for the Global Burden of Disease Study 2021. Lancet. (2024) 403(10440):2133–2161. doi: 10.1016/S0140-6736(24)00757-8 PMC11122111 38642570

[3] Di Nisio M van Es N Büller HR . Deep vein thrombosis and pulmonary embolism. Lancet. (2016) 388:3060–73. doi: 10.1016/s0140-6736(16)30514-1 27375038

[4] Woo KS Tse LKK Tse CY Metreweli C Vallance-Owen J . The prevalence and pattern of pulmonary thromboembolism in the Chinese in Kong Kong. Int J Cardiol. (1988) 20:373–80. doi: 10.1016/0167-5273(88)90291-4 3262592

[5] Holst AG Jensen G Prescott E . Risk factors for venous thromboembolism. Circulation. (2010) 121:1896–903. doi: 10.1161/circulationaha.109.921460 20404252

[6] Cohen O Santagata D Ageno W . Novel horizons in anticoagulation: the emerging role of factor XI inhibitors across different settings. Haematologica. (2024) 109:3110–24. doi: 10.3324/haematol.2023.283682 38779744 PMC11443408

[7] Perera V Wang Z Luettgen J Li D DeSouza M Cerra M . First‐in‐human study of milvexian, an oral, direct, small molecule factor XIa inhibitor. Clin Transl Sci. (2021) 15:330–42. doi: 10.1111/cts.13148 34558200 PMC8841437

[8] Perera V Wang Z Lubin S Ueno T Shiozaki T Chen W . Safety, pharmacokinetics, and pharmacodynamics of milvexian in healthy Japanese participants. Sci Rep. (2022) 12:5165. doi: 10.1038/s41598-022-08768-y 35338177 PMC8956633

[9] Zivelin A Varon D Zucker M Steinberg D Salomon O Seligsohn U . Patients with severe factor XI deficiency have a reduced incidence of deep-vein thrombosis. Thromb Haemostasis. (2011) 105:269–73. doi: 10.1160/th10-05-0307 21057700

[10] Büller HR Bethune C Bhanot S Gailani D Monia BP Raskob GE . Factor XI antisense oligonucleotide for prevention of venous thrombosis. N Engl J Med. (2015) 372:232–40. doi: 10.1182/blood.v124.21.lba-1.lba-1 PMC4367537 25482425

[11] Löwenberg EC Meijers JCM Monia BP Levi M . Coagulation factor XI as a novel target for antithrombotic treatment. J Thromb Haemostasis. (2010) 8:2349–57. doi: 10.1111/j.1538-7836.2010.04031.x 20727068

[12] Greco A Laudani C Spagnolo M Agnello F Faro DC Finocchiaro S . Pharmacology and clinical development of factor XI inhibitors. Circulation. (2023) 147:897–913. doi: 10.1161/circulationaha.122.062353 36913497

[13] Fujikawa K Chung DW Hendrickson LE Davie EW . Amino acid sequence of human factor XI, a blood coagulation factor with four tandem repeats that are highly homologous with plasma prekallikrein. Biochemistry. (2002) 25:2417–24. doi: 10.1021/bi00357a018 3636155

[14] Moellmer SA Puy C McCarty OJT . Biology of factor XI. Blood. (2024) 143:1445–54. doi: 10.1182/blood.2023020719 37874916 PMC11033592

[15] Papagrigoriou E McEwan PA Walsh PN Emsley J . Crystal structure of the factor XI zymogen reveals a pathway for transactivation. Nat Struct Mol Biol. (2006) 13:557–8. doi: 10.1038/nsmb1095 16699514

[16] Baglia FA Walsh PN . A binding site for thrombin in the Apple 1 domain of factor XI. J Biol Chem. (1996) 271:3652–8. doi: 10.1074/jbc.271.7.3652 8631976

[17] Ho DH Badellino K Baglia FA Sun MF Zhao MM Gailani D . The role of high molecular weight kininogen and prothrombin as cofactors in the binding of factor XI A3 domain to the platelet surface. J Biol Chem. (2000) 275:25139–45. doi: 10.1074/jbc.m001890200 10823824

[18] Renné T Gailani D Meijers JCM Müller-Esterl W . Characterization of the H-kininogen-binding site on factor XI. J Biol Chem. (2002) 277:4892–9. doi: 10.1074/jbc.M105221200 11733491

[19] Sun M-F Zhao M Gailani D . Identification of amino acids in the factor XI Apple 3 domain required for activation of factor IX. J Biol Chem. (1999) 274:36373–8. doi: 10.1074/jbc.274.51.36373 10593931

[20] Baglia FA Gailani D López JA Walsh PN . Identification of a binding site for glycoprotein Ibα in the Apple 3 domain of factor XI. J Biol Chem. (2004) 279:45470–6. doi: 10.1074/jbc.m406727200 15317813

[21] Ho DH Badellino K Baglia FA Walsh PN . A binding site for heparin in the Apple 3 domain of factor XI. J Biol Chem. (1998) 273:16382–90. doi: 10.1074/jbc.273.26.16382 9632702

[22] Zhao M Abdel-Razek T Sun MF Gailani D . Characterization of a heparin binding site on the heavy chain of factor XI. J Biol Chem. (1998) 273:31153–9. doi: 10.1074/jbc.273.47.31153 9813019

[23] Baglia FA Jameson Ba Fau - Walsh PN Walsh PN . Identification and characterization of a binding site for factor XIIa in the Apple 4 domain of coagulation factor XI. J Biol Chem. (1993) 268:3838–44. doi: 10.1016/s0021-9258(18)53549-3 8440679

[24] Wu W Sinha D Shikov S Yip CK Walz T Billings PC . Factor XI homodimer structure is essential for normal proteolytic activation by factor XIIa, thrombin, and factor XIa. J Biol Chem. (2008) 283:18655–64. doi: 10.1074/jbc.m802275200 18441012 PMC2441546

[25] Zucker M Zivelin A Landau M Rosenberg N Seligsohn U . Three residues at the interface of factor XI (FXI) monomers augment covalent dimerization of FXI. J Thromb Haemostasis. (2009) 7:970–5. doi: 10.1111/j.1538-7836.2009.03353.x 19548906

[26] Al-Horani RA Afosah DK . Recent advances in the discovery and development of factor XI/XIa inhibitors. Med Res Rev. (2018) 38:1974–2023. doi: 10.1002/med.21503 29727017 PMC6173998

[27] Hsu C Hutt E Bloomfield DM Gailani D Weitz JI . Factor XI inhibition to uncouple thrombosis from hemostasis. J Am Coll Cardiol. (2021) 78:625–31. doi: 10.1016/j.jacc.2021.06.010 34353538 PMC8552338

[28] Puy C Tucker EI Matafonov A Cheng Q Zientek KD Gailani D . Activated factor XI increases the procoagulant activity of the extrinsic pathway by inactivating tissue factor pathway inhibitor. Blood. (2015) 125:1488–96. doi: 10.1182/blood-2014-10-604587 PMC4342360 25587039

[29] Smith S Choi S Morrissey J . Polyphosphate accelerates factor V activation by factor XIa. Thromb Haemostasis. (2017) 113:599–604. doi: 10.1160/th14-06-0515 25338662 PMC4541292

[30] Garland KS Reitsma SE Shirai T Zilberman-Rudenko J Tucker EI Gailani D . Removal of the C-terminal domains of ADAMTS13 by activated coagulation factor XI induces platelet adhesion on endothelial cells under flow conditions. Front Med. (2017) 4:232. doi: 10.3389/fmed.2017.00232 29326937 PMC5742325

[31] Al-Horani RA Desai UR . Factor XIa inhibitors: a review of the patent literature. Expert Opin Ther Pat. (2016) 26:323–45. doi: 10.1517/13543776.2016.1154045 26881476 PMC4941829

[32] Smith SA Travers RJ Morrissey JH . How it all starts: initiation of the clotting cascade. Crit Rev Biochem Mol Biol. (2015) 50:326–36. doi: 10.3109/10409238.2015.1050550 26018600 PMC4826570

[33] Whelihan MF Orfeo T Gissel MT Mann KG . Coagulation procofactor activation by factor XIa. J Thromb Haemostasis. (2010) 8:1532–9. doi: 10.1111/j.1538-7836.2010.03899.x 20456758 PMC2909362

[34] Matafonov A Cheng Q Geng Y Verhamme IM Umunakwe O Tucker EI . Evidence for factor IX‐independent roles for factor XIa in blood coagulation. J Thromb Haemostasis. (2013) 11:2118–27. doi: 10.1111/jth.12435 24152424 PMC3947433

[35] Geng Y Verhamme IM Smith SB Sun Mf Matafonov A Cheng Q . The dimeric structure of factor XI and zymogen activation. Blood. (2013) 121:3962–9. doi: 10.1182/blood-2012-12-473629 23515926 PMC3650707

[36] Bouma BN Meijers JCM . Role of blood coagulation factor XI in downregulation of fibrinolysis. Curr Opin Hematol. (2000) 7:266–72. doi: 10.1097/00062752-200009000-00002 10961575

[37] Engelmann B Massberg S . Thrombosis as an intravascular effector of innate immunity. Nat Rev Immunol. (2013) 13:34–45. doi: 10.1038/nri3345 23222502

[38] McFadyen JD Stevens H Peter K . The emerging threat of (micro)thrombosis in COVID-19 and its therapeutic implications. Circ Res. (2020) 127:571–87. doi: 10.1161/circresaha.120.317447 32586214 PMC7386875

[39] Esmon CT Xu J Lupu F . Innate immunity and coagulation. J Thromb Haemostasis. (2011) 9:182–8. doi: 10.1111/j.1538-7836.2011.04323.x PMC3151110 21781254

[40] Seong S-Y Matzinger P . Hydrophobicity: an ancient damage-associated molecular pattern that initiates innate immune responses. Nat Rev Immunol. (2004) 4:469–78. doi: 10.1038/nri1372 15173835

[41] van der Poll T Shankar-Hari M Wiersinga WJ . The immunology of sepsis. Immunity. (2021) 54:2450–64. doi: 10.1016/j.immuni.2021.10.012 34758337

[42] Ma AC Kubes P . Platelets, neutrophils, and neutrophil extracellular traps (NETs) in sepsis. J Thromb Haemostasis. (2008) 6:415–20. doi: 10.1111/j.1538-7836.2007.02865.x 18088344

[43] Stark K Philippi V Stockhausen S Busse J Antonelli A Miller M . Disulfide HMGB1 derived from platelets coordinates venous thrombosis in mice. Blood. (2016) 128:2435–49. doi: 10.1182/blood-2016-04-710632 27574188 PMC5147023

[44] Itakura A Verbout NG Phillips KG Insall RH Gailani D Tucker EI . Activated factor XI inhibits chemotaxis of polymorphonuclear leukocytes. J Leuk Biol. (2011) 90:923–7. doi: 10.1189/jlb.0411182 21807745 PMC3206472

[45] Xue M Wang S Li C Wang Y Liu M Huang X . Deficiency of neutrophil gelatinase-associated lipocalin elicits a hemophilia-like bleeding and clotting disorder in mice. Blood. (2025) 145:975–87. doi: 10.1182/blood.2024026476 39693621

[46] Pixley RA De La Cadena R Page JD Kaufman N Wyshock EG Chang A . The contact system contributes to hypotension but not disseminated intravascular coagulation in lethal bacteremia. *In vivo* use of a monoclonal anti-factor XII antibody to block contact activation in baboons. J Clin Invest. (1993) 91:61–8. doi: 10.1172/jci116201 7678610 PMC329995

[47] Puy C Pang J Reitsma SE Lorentz CU Tucker EI Gailani D . Cross-talk between the complement pathway and the contact activation system of coagulation: activated factor XI neutralizes complement factor H. J Immunol. (2021) 206:1784–92. doi: 10.4049/jimmunol.2000398 33811105 PMC8030746

[48] Puy C Pang J Reitsma SE Lorentz CU Tucker EI Gailani D . Platelets and neutrophil extracellular traps collaborate to promote intravascular coagulation during sepsis in mice. Blood. (2017) 129:1357–67. doi: 10.1182/blood-2016-09-741298 28073784 PMC5345735

[49] Skendros P Mitsios A Chrysanthopoulou A Mastellos DC Metallidis S Rafailidis P . Complement and tissue factor–enriched neutrophil extracellular traps are key drivers in COVID-19 immunothrombosis. J Clin Invest. (2020) 130:6151–7. doi: 10.1172/jci141374 32759504 PMC7598040

[50] Heal SL Hardy LJ Wilson CL Ali M Ariëns RAS Foster R . Novel interaction of properdin and coagulation factor XI: crosstalk between complement and coagulation. Res Pract Thromb Haemostasis. (2022) 6:e12715. doi: 10.1002/rth2.12715 35647477 PMC9130567

[51] Bane CE Jr Ivanov I Matafonov A Boyd KL Cheng Q Sherwood ER . Factor XI deficiency alters the cytokine response and activation of contact proteases during polymicrobial sepsis in mice. PloS One. (2016) 11:e0152968. doi: 10.1371/journal.pone.0152968 PMC4821616 27046148

[52] Visser M Heitmeier S Ten Cate H Spronk HMH . Role of factor XIa and plasma kallikrein in arterial and venous thrombosis. Thromb Haemost. (2020) 120:883–993. doi: 10.1055/s-0040-1710013 32375196

[53] Schmaier AH . The contact activation and kallikrein/kinin systems: pathophysiologic and physiologic activities. J Thromb Haemost. (2016) 14:28–39. doi: 10.1111/jth.13194 26565070

[54] Mauron T Lammle B Wuillemin WA . High molecular weight kininogen is cleaved by FXIa at three sites: Arg409-Arg410, Lys502-Thr503 and Lys325-Lys326. Thromb Haemost. (2000) 83:709–14. doi: 10.1055/s-0037-1613897 10823267

[55] Yamaguchi Y Morser J Leung LL . Chemerin bioactivity is regulated by factor XIa: a novel interface linking between coagulation, hemostasis and immunity. Blood. (2011) 118:2258. doi: 10.1182/blood.v118.21.2258.2258

[56] Shi T Giannakopoulos B Iverson GM Cockerill KA Linnik MD Krilis SA . Domain V of β2-glycoprotein I binds factor XI/XIa and is cleaved at Lys317-Thr318. J Biol Chem. (2005) 280:907–12. doi: 10.1074/jbc.m410291200 15522884

[57] Ge X Yamaguchi Y Zhao L Bury L Gresele P Berube C . Prochemerin cleavage by factor XIa links coagulation and inflammation. Blood. (2018) 131:353–64. doi: 10.1182/blood-2017-07-792580 29158361 PMC5774209

[58] Li Y Bezemer ID Rowland CM Tong CH Arellano AR Catanese JJ . Genetic variants associated with deep vein thrombosis: the F11 locus. J Thromb Haemostasis. (2009) 7:1802–8. doi: 10.1111/j.1538-7836.2009.03544.x 19583818

[59] Lärfars G Hamsten A Kieler H Silveira A Holmström M Strawbridge RJ . F11 is associated with recurrent VTE in women. Thromb Haemostasis. (2017) 115:406–14. doi: 10.1160/TH15-06-0459 26423325

[60] Song N Tian Ax Zhang Jm Jiang Hq Zang Jc Ma Xl . F11 rs2289252T and rs2036914C polymorphisms increase the activity of factor XI in post‐trauma patients with fractures despite thromboprophylaxis. Orthopaedic Surg. (2016) 8:377–82. doi: 10.1111/os.12262 27627722 PMC6584134

[61] Van Laer C Peerlinck K Jacquemin M Thys C Downes K Labarque V . F11 gene duplication causes elevated FXI plasma levels and is a risk for venous thrombosis. Thromb Haemostasis. (2021) 122:1058–60. doi: 10.1055/s-0041-1739363 34781376

[62] Preis M Hirsch J Kotler A Zoabi A Stein N Rennert G . Factor XI deficiency is associated with lower risk for cardiovascular and venous thromboembolism events. Blood. (2017) 129:1210–5. doi: 10.1182/blood-2016-09-742262 28039189

[63] El-Maarri O Hanson E Nilsson S Jood K Norrving B Engström G . Genetic variants of coagulation factor XI show association with ischemic stroke up to 70 years of age. PloS One. (2013) 8:e75286. doi: 10.1371/journal.pone.0075286 PMC3783404 24086496

[64] Gill D Georgakis MK Laffan M Sabater-Lleal M Malik R Tzoulaki I . Genetically determined FXI (factor XI) levels and risk of stroke. Stroke. (2018) 49:2761–3. doi: 10.1161/strokeaha.118.022792 30355187

[65] Bare LA Arellano AR Tong CH Bezemer I Lijfering WM Devlin JJ . Genetic variants of F11, statin use and venous thrombosis. J Thromb Haemostasis. (2011) 9:1249–51. doi: 10.1111/j.1538-7836.2011.04289.x 21486383

[66] Campo G Lunghi B Pavasini R Ferraresi P Punzetti S Malagù M . Factor XI rs2036914 gene polymorphism and occurrence of adverse events after percutaneous coronary intervention. A prospective evaluation. Int J Cardiol. (2014) 177:711–3. doi: 10.1016/j.ijcard.2014.10.014 25456690

[67] Gagnon E Girard A Bourgault J Abner E Gill D Thériault S . Genetic assessment of efficacy and safety profiles of coagulation cascade proteins identifies factors II and XI as actionable anticoagulant targets. Eur Heart J Open. (2024) 4:oeae043. doi: 10.1093/ehjopen/oeae043 38933427 PMC11200102

[68] Sharma M Dong Q Hirano T Kasner SE Saver JL Masjuan J . Asundexian for secondary stroke prevention. N Engl J Med. (2026) 394:1467–79. doi: 10.1056/nejmoa2513880 41985132

[69] Piccini JP Patel MR Steffel J Ferdinand K Van Gelder IC Russo AM . Asundexian versus apixaban in patients with atrial fibrillation. N Engl J Med. (2025) 392:23–32. doi: 10.1056/nejmoa2407105 39225267

[70] Heitmeier S Visser M Tersteegen A Dietze-Torres J Glunz J Gerdes C . Pharmacological profile of asundexian, a novel, orally bioavailable inhibitor of factor XIa. J Thromb Haemostasis. (2022) 20:1400–11. doi: 10.1111/jth.15700 35289054 PMC9313898

[71] Chen H Hashizume K Kanefendt F Brase C Schmitz S Liu T . Pharmacokinetics, pharmacodynamics, and safety of asundexian in healthy Chinese and Japanese volunteers, and comparison with Caucasian data. Clin Transl Sci. (2024) 17:e13895. doi: 10.1111/cts.13895 39082898 PMC11289896

[72] Rao SV Kirsch B Bhatt DL Budaj A Coppolecchia R Eikelboom J . A multicenter, phase 2, randomized, placebo-controlled, double-blind, parallel-group, dose-finding trial of the oral factor XIa inhibitor asundexian to prevent adverse cardiovascular outcomes after acute myocardial infarction. Circulation. (2022) 146:1196–206. doi: 10.1161/circulationaha.122.061612 36030390

[73] Shoamanesh A Mundl H Smith EE Masjuan J Milanov I Hirano T . Factor XIa inhibition with asundexian after acute non-cardioembolic ischaemic stroke (PACIFIC-Stroke): an international, randomised, double-blind, placebo-controlled, phase 2b trial. Lancet. (2022) 400:997–1007. doi: 10.1016/s0140-6736(22)01588-4 36063821

[74] Khatri P Shoamanesh A Mundl H Smith E Saad F Xu L . Abstract WP177: Effects of the oral factor XIa inhibitor asundexian on intracranial bleeding among patients with acute non-cardioembolic ischemic stroke: PACIFIC-Stroke randomized trial. Stroke. (2023) 54(Suppl_1):AWP177–AWP177. doi: 10.1161/str.54.suppl_1.wp177

[75] Balali P Hart RG Smith EE Saad F Colorado P Lemmens R . Cerebral microbleeds and asundexian in non-cardioembolic ischemic stroke: secondary analyses of the PACIFIC-STROKE randomized trial. Int J Stroke. (2023) 19:526–35. doi: 10.1177/17474930231216339 37950392 PMC11134999

[76] Smith EE Shoamanesh A Xu L Heenan L Saad F Colorado P . Effect of the factor XIa inhibitor asundexian according to baseline infarct pattern and on MRI covert infarct outcomes. Stroke. (2024) 55:392–402. doi: 10.1161/strokeaha.123.043198 38174569

[77] Piccini JP Caso V Connolly SJ Fox KAA Oldgren J Jones WS . Safety of the oral factor XIa inhibitor asundexian compared with apixaban in patients with atrial fibrillation (PACIFIC-AF): a multicentre, randomised, double-blind, double-dummy, dose-finding phase 2 study. Lancet. (2022) 399:1383–90. doi: 10.1016/s0140-6736(22)00456-1 35385695

[78] Alexander JH Lydon EJ Piccini JP Viethen T Oldgren J Goodman SG . Asundexian or apixaban in patients with atrial fibrillation according to prior oral anticoagulant use. JAMA Cardiol. (2025) 10:555–63. doi: 10.1001/jamacardio.2025.0277 40136309 PMC11947966

[79] Pollack CV Kurz MA Hayward NJ . EP-7041, a factor XIa inhibitor as a potential antithrombotic strategy in extracorporeal membrane oxygenation: a brief report. Crit Care Explor. (2020) 2:e0196. doi: 10.1097/cce.0000000000000196 32984829 PMC7498136

[80] Zhang Jy Ruan Zr Jiang B Yang Dd Wang Jy Hu Y . Pharmacokinetics, pharmacodynamics, and safety of frunexian in healthy Chinese volunteer adults: a randomized dose‐escalation phase I study. Clin Transl Sci. (2024) 17:e13787. doi: 10.1111/cts.13787 38558535 PMC10983022

[81] Letunica N Karlaftis V Monagle P Patino H Chintala M Attard C . The *in vitro* anticoagulant effect of milvexian in healthy neonates and children. Thromb Res. (2025) 257:109564. doi: 10.1016/j.thromres.2025.109564 41406613

[82] Dilger AK Pabbisetty KB Corte JR De Lucca I Fang T Yang W . Discovery of milvexian, a high-affinity, orally bioavailable inhibitor of factor XIa in clinical studies for antithrombotic therapy. J Med Chem. (2021) 65:1770–85. doi: 10.1021/acs.jmedchem.1c00613 34494428

[83] Wong PC Crain EJ Bozarth JM Wu Y Dilger AK Wexler RR . Milvexian, an orally bioavailable, small‐molecule, reversible, direct inhibitor of factor XIa: *in vitro* studies and *in vivo* evaluation in experimental thrombosis in rabbits. J Thromb Haemostasis. (2022) 20:399–408. doi: 10.1111/jth.15588 34752670 PMC9299130

[84] Luo Z Wang J Niu Z Hu C Chintala M Luo X . Pharmacokinetics, pharmacodynamics, safety, and tolerability of milvexian in healthy Chinese adults. Drug Des Dev Ther. (2025) 19:1503–14. doi: 10.2147/dddt.s488414 40045992 PMC11881624

[85] Jarugula P Soleman S Back H Christopher LJ Hawthorne D Aronson R . Absolute oral bioavailability of milvexian spray‐dried dispersion formulation under fasted and fed conditions in healthy adult participants: an intravenous microtracer approach. Clin Transl Sci. (2024) 17:e70058. doi: 10.1111/cts.70058 39450784 PMC11503494

[86] Perera V Abelian G Li D Wang Z Zhang L Lubin S . Single-dose pharmacokinetics of milvexian in participants with mild or moderate hepatic impairment compared with healthy participants. Clin Pharmacokinet. (2022) 61:857–67. doi: 10.1007/s40262-022-01110-9 35262846 PMC9249726

[87] Perera V Abelian G Li D Wang Z Zhang L Lubin S . Single-dose pharmacokinetics of milvexian in participants with normal renal function and participants with moderate or severe renal impairment. Clin Pharmacokinet. (2022) 61:1405–16. doi: 10.1007/s40262-022-01150-1 35906349 PMC9553801

[88] Perera V Abelian G Luettgen J Aronson R Li D Wang Z . A randomized, cross-over study to evaluate the safety, tolerability, pharmacokinetics, and pharmacodynamics of milvexian with single and dual antiplatelet therapy in healthy participants. European Heart Journal. (2022) 43(Supplement_2):ehac544.2704. doi: 10.1093/eurheartj/ehac544.2704

[89] Perera V Abelian G Luettgen J Aronson R Li D Wang Z . Safety, tolerability, pharmacokinetics and pharmacodynamics of milvexian with aspirin and/or clopidogrel in healthy participants. Sci Rep. (2024) 14:16591. doi: 10.1038/s41598-024-67182-8 39025971 PMC11258331

[90] Weitz JI Strony J Ageno W Gailani D Hylek EM Lassen MR . Milvexian for the prevention of venous thromboembolism. N Engl J Med. (2021) 385:2161–72. doi: 10.1056/nejmoa2113194 34780683 PMC9540352

[91] Sharma M Molina CA Toyoda K Bereczki D Bangdiwala SI Kasner SE . Safety and efficacy of factor XIa inhibition with milvexian for secondary stroke prevention (AXIOMATIC-SSP): a phase 2, international, randomised, double-blind, placebo-controlled, dose-finding trial. Lancet Neurol. (2024) 23:46–59. doi: 10.1016/s1474-4422(23)00403-9 38101902 PMC10822143

[92] Pinto DJP Orwat MJ Smith LM Quan ML Lam PYS Rossi KA . Discovery of a parenteral small molecule coagulation factor XIa inhibitor clinical candidate (BMS-962212). J Med Chem. (2017) 60:9703–23. doi: 10.1021/acs.jmedchem.7b01171 29077405

[93] Perera V Luettgen JM Wang Z Frost CE Yones C Russo C . First‐in‐human study to assess the safety, pharmacokinetics and pharmacodynamics of BMS‐962212, a direct, reversible, small molecule factor XIa inhibitor in non‐Japanese and Japanese healthy subjects. Br J Clin Pharmacol. (2018) 84:876–87. doi: 10.1111/bcp.13520 29346838 PMC5903252

[94] Chen R Guan X Hu P Dong Y Zhu Y Zhang T . First-in-human study to assess the safety, pharmacokinetics, and pharmacodynamics of SHR2285, a small-molecule factor XIa inhibitor in healthy subjects. Front Pharmacol. (2022) 13:821363. doi: 10.3389/fphar.2022.821363 35222036 PMC8866703

[95] Xu J Zhao N Huang J Li J Zhao X Xiang Q . The safety, pharmacokinetics, and pharmacodynamics of SHR2285, an oral small molecule factor XIa inhibitor, in healthy Chinese volunteers. Clin Drug Invest. (2023) 43:435–45. doi: 10.1007/s40261-023-01281-8 37326942

[96] Ma T Dong Y Huang L Yang Y Geng Y Fei F . SHR2285, the first selectively oral FXIa inhibitor in China: safety, tolerability, pharmacokinetics and pharmacodynamics combined with aspirin, clopidogrel or ticagrelor. Front Pharmacol. (2022) 13:1027627. doi: 10.3389/fphar.2022.1027627 36339534 PMC9626527

[97] Weitz JI Chan NC . MAA868 locks factor XIa in a zymogen-like state. Blood. (2019) 133:1393–4. doi: 10.1182/blood-2019-02-900480 30923107

[98] Koch AW Schiering N Melkko S Ewert S Salter J Zhang Y . MAA868, a novel FXI antibody with a unique binding mode, shows durable effects on markers of anticoagulation in humans. Blood. (2019) 133:1507–16. doi: 10.1182/blood-2018-10-880849 30692123

[99] Yi BA Freedholm D Widener N Wang X Simard E Cullen C . Pharmacokinetics and pharmacodynamics of abelacimab (MAA868), a novel dual inhibitor of factor XI and factor XIa. J Thromb Haemostasis. (2022) 20:307–15. doi: 10.1111/jth.15577 34714969 PMC9298689

[100] Verhamme P Yi BA Segers A Salter J Bloomfield D Büller HR . Abelacimab for prevention of venous thromboembolism. N Engl J Med. (2021) 385:609–17. doi: 10.1056/nejmoa2105872 34297496

[101] Ruff CT Patel SM Giugliano RP Morrow DA Hug B Kuder JF . Abelacimab versus rivaroxaban in patients with atrial fibrillation. N Engl J Med. (2025) 392:361–71. doi: 10.1056/nejmoa2406674 39842011

[102] Al Said S Patel SM Giugliano RP Morrow DA Goodrich EL Murphy SA . Abelacimab versus rivaroxaban in patients with atrial fibrillation on antiplatelet therapy: A prespecified analysis of the AZALEA-TIMI 71 trial. Circulation. (2025) 152:290–6. doi: 10.1161/circulationaha.125.074037 40546068 PMC12668221

[103] Zeng S Kaisaier W Liu C Zhu W . Factor XI/XIa inhibitors versus direct oral anticoagulants in atrial fibrillation. Eur J Internal Med. (2025) 137:140–1. doi: 10.1016/j.ejim.2025.02.013 39929757

[104] Schaefer M Buchmueller A Dittmer F Straßburger J Wilmen A . Allosteric inhibition as a new mode of action for BAY 1213790, a neutralizing antibody targeting the activated form of coagulation factor XI. J Mol Biol. (2019) 431:4817–33. doi: 10.1016/j.jmb.2019.09.008 31655039

[105] Samaha E Schwameis M Schranz S Kovacevic KD Watschinger B Stoiber M . The factor XIa antibody osocimab strongly inhibits clotting in extracorporeal circuits with human blood and in baboons. Res Pract Thromb Haemost. (2025) 9 (4):102932. doi: 10.1016/j.rpth.2025.102932 PMC12275933 40687384

[106] Thomas D Thelen K Kraff S Schwers S Schiffer S Unger S . BAY 1213790, a fully human IgG1 antibody targeting coagulation factor XIa: First evaluation of safety, pharmacodynamics, and pharmacokinetics. Res Pract Thromb Haemostasis. (2019) 3:242–53. doi: 10.1002/rth2.12186 31011708 PMC6462747

[107] Weitz JI Bauersachs R Becker B Berkowitz SD Freitas MCS Lassen MR . Effect of osocimab in preventing venous thromboembolism among patients undergoing knee arthroplasty. Jama. (2020) 323:130–9. doi: 10.1001/jama.2019.20687 31935028 PMC6990695

[108] Weitz JI Tankó LB Floege J Fox KAA Bhatt DL Thadhani R . Anticoagulation with osocimab in patients with kidney failure undergoing hemodialysis: A randomized phase 2 trial. Nat Med. (2024) 30:435–42. doi: 10.1038/s41591-023-02794-7 38365952 PMC10878964

[109] Leung PY Hurst S Berny-Lang MA Verbout NG Gailani D Tucker EI . Inhibition of factor XII-mediated activation of factor XI provides protection against experimental acute ischemic stroke in mice. Trans Stroke Res. (2012) 3:381–9. doi: 10.1007/s12975-012-0186-5 23634198 PMC3637928

[110] Cheng Q Tucker EI Pine MS Sisler I Matafonov A Sun Mf . A role for factor XIIa–mediated factor XI activation in thrombus formation *in vivo*. Blood. (2010) 116:3981–9. doi: 10.1182/blood-2010-02-270918 20634381 PMC2981546

[111] Lorentz CU Verbout NG Wallisch M Hagen MW Shatzel JJ Olson SR . Contact activation inhibitor and factor XI antibody, AB023, produces safe, dose-dependent anticoagulation in a phase 1 first-in-human trial. Arterioscler Thromb Vasc Biol. (2019) 39:799–809. doi: 10.1161/atvbaha.118.312328 30700130 PMC6494446

[112] Lorentz CU Verbout NG Shatzel JJ Wallisch M Markway BD Hinds MT . Safety and efficacy of the contact activation inhibitor AB023 in patients with end-stage renal disease on chronic hemodialysis: A phase 2, double-blind, randomized, placebo-controlled trial. Blood. (2020) 136:23–4. doi: 10.1182/blood-2020-139948

[113] Pfeffer MA Kohs TCL Vu HH Jordan KR Wang JSH Lorentz CU . Factor XI inhibition for the prevention of catheter-associated thrombosis in patients with cancer undergoing central line placement: A phase 2 clinical trial. Arterioscler Thromb Vasc Biol. (2024) 44:290–9. doi: 10.1161/atvbaha.123.319692 PMC10877270 37970718

[114] Ma T Weng Z Cao B Dong Y Deng C Huang L . The first-in-human study to assess the safety, tolerability, pharmacokinetics, and pharmacodynamics of the factor XI monoclonal antibody SHR-2004 in healthy subjects. Expert Opin Invest Drugs. (2024) 33:1075–82. doi: 10.1080/13543784.2024.2391837 39166425

[115] Xiao W Chai W Xie D Kong X Nan S Kang P . Efficacy and safety of SHR-2004 for venous thromboembolism prevention after elective total knee arthroplasty: results from a randomized, double-blind, active-controlled phase 3 study. In: ISTH 2026 Congress. Paris. International Society on Thrombosis and Haemostasis. (2026). Abstract LB 01.2.

[116] Nowotny B Thomas D Schwers S Wiegmann S Prange W Yassen A . First randomized evaluation of safety, pharmacodynamics, and pharmacokinetics of BAY 1831865, an antibody targeting coagulation factor XI and factor XIa, in healthy men. J Thromb Haemostasis. (2022) 20:1684–95. doi: 10.1111/jth.15744 35490404 PMC9320929

[117] Xiang Q Liu Z Xie Q Zhao N Zhou S Cao L . Pharmacokinetics, pharmacodynamics and safety profile of SKB336, a selective inhibitor of coagulation factor XI/XIa in healthy subjects. Br J Clin Pharmacol. (2025) 91:2817–26. doi: 10.1002/bcp.70043 40204290

[118] He X Zhang J Du Y Liu X Hu D Cao B . BJTJ-1837, a novel FXI activation-blocking antibody. Res Pract Thromb Haemostasis. (2023) 7:100067. doi: 10.1016/j.rpth.2023.100067 36936858 PMC10017418

[119] Monia BP Black C Gao D Siwkowski A Watt A Freier SM . Antithrombotic effects of a FXI antisense oligonucleotide in mice. Blood. (2008) 112:3076. doi: 10.1182/blood.v112.11.3076.3076

[120] Zhang H Lowenberg EC Crosby JR MacLeod AR Zhao C Gao D . Inhibition of the intrinsic coagulation pathway factor XI by antisense oligonucleotides: a novel antithrombotic strategy with lowered bleeding risk. Blood. (2010) 116 (22):4684–92. doi: 10.1182/blood-2010-04-277798 20807891

[121] Younis H Crosby JR Zhao C MacLeod R Monia BP Henry S . ISIS-FXIRx, a potent antisense inhibitor of factor XI, suppresses FXI activity and elevates Aptt without increased risk of bleeding in cynomolgus monkeys following 13 weeks of treatment. Blood. (2011) 118:2255. doi: 10.1182/blood.v118.21.2255.2255

[122] Crosby JR Marzec UM Zhao C Gao D Revenko AS Matafonov A . Antisense oligonucleotide mediated depletion of factor XI prevents vascular occlusion in an experimental thrombosis model in primates. Blood. (2011) 118:2250. doi: 10.1182/blood.v118.21.2250.2250

[123] Yau JW Liao P Fredenburgh JC Stafford AR Revenko AS Monia BP . Selective depletion of factor XI or factor XII with antisense oligonucleotides attenuates catheter thrombosis in rabbits. Blood. (2014) 123:2102–7. doi: 10.1182/blood-2013-12-540872 24501216

[124] Walsh M Bethune C Smyth A Tyrwhitt J Jung SW Yu RZ . Phase 2 study of the factor XI antisense inhibitor IONIS-FXIRx in patients with ESRD. Kidney Int Rep. (2022) 7:200–9. doi: 10.1016/j.ekir.2021.11.011 35155859 PMC8820988

[125] Bethune C Morgan E Schulz D Yu RZ Jung SW Geary RS . IONIS-FXI-LRx, an FXI GalNAc-conjugated antisense drug, produces potent and sustained reduction in FXI activity in normal volunteers. Poster presented at: DIA/FDA Oligonucleotide-Based Therapeutics Conference. North Bethesda, MD. (2019) 28–30.

[126] Liu T Hashizume K Krieg E Chen H Mukaida Y Thelen K . Pharmacokinetics, pharmacodynamics, and safety of fesomersen, a novel antisense inhibitor of factor XI, in healthy Chinese, Japanese, and Caucasian volunteers. Clin Transl Sci. (2024) 17:e13784. doi: 10.1111/cts.13784 38563414 PMC10985948

[127] Winkelmayer WC Lensing AWA Thadhani RI Mahaffey KW Walsh M Pap ÁF . A phase II randomized controlled trial evaluated antithrombotic treatment with fesomersen in patients with kidney failure on hemodialysis. Kidney Int. (2024) 106:145–53. doi: 10.1016/j.kint.2024.02.024 38537676

[128] Liang J Nilsson S Wikström J Cao H Zheng S Xu Q . Inhibition of factor XI using RBD4059. JACC: Basic to Trans Sci. (2025) 10:786–97. doi: 10.1016/j.jacbts.2024.12.005 40562491 PMC12230477

[129] Yue P Qu E Pan J Weitz JI Ji Q . A phase 1, single ascending dose study to evaluate the safety, tolerability, pharmacokinetics, and pharmacodynamics of subcutaneously administered SRSD107 in healthy subjects. Blood. (2024) 144:4009. doi: 10.1182/blood-2024-203718

[130] Ye X Deng X Gao H Chen S Ding L Sun F . Functional characterization of a common XIa inhibitory Kunitz-domain Shp4 from nine schistosoma secreted proteins with diverse C-terminal fragments. PloS Negl Trop Dis. (2025) 19:e0013282. doi: 10.1371/journal.pntd.0013282 PMC12237034 40627652

[131] Smith RP Higuchi DA Broze GJ . Platelet coagulation factor XI a -inhibitor, a form of Alzheimer amyloid precursor protein. Science. (1990) 248:1126–8. doi: 10.1126/science.2111585 2111585

[132] Wu W Li H Navaneetham D Reichenbach ZW Tuma RF Walsh PN . The kunitz protease inhibitor domain of protease nexin-2 inhibits factor XIa and murine carotid artery and middle cerebral artery thrombosis. Blood. (2012) 120:671–7. doi: 10.1182/blood-2012-03-419523 22674803 PMC3401218

[133] Chng WS Li AWL Lim JJM Leong EJE Amran FS Kini RM . A factor XIa inhibitor engineered from banded krait venom toxin: Efficacy and safety in rodent models of arterial and venous thrombosis. Biomedicines. (2022) 10:1679. doi: 10.3390/biomedicines10071679 35884984 PMC9312835

[134] Sun F Wang W Li Z Li Y Guo W Kong Y . Design, expression and biological evaluation of DX-88mut as a novel selective factor XIa inhibitor for antithrombosis. Bioorg Chem. (2024) 142:106951. doi: 10.1016/j.bioorg.2023.106951 37924755

[135] Dai L Qiu Y Xu Q Yang F Ren B Zhuang X . Discovery of potent and selective factor XIa inhibitors incorporating triazole-based benzoic acid as novel P2’ fragments: Molecular dynamics simulations and anticoagulant activity. Eur J Med Chem. (2025) 282:117067. doi: 10.1016/j.ejmech.2024.117067 39580913

[136] Zhang Y Huang R Hu X Zheng N Geng L Yin Z . Discovery of potent, highly selective, and orally bioavailable factor XIa inhibitors for anticoagulant therapy. Eur J Med Chem. (2025) 289:117436. doi: 10.1016/j.ejmech.2025.117436 40010270

[137] Wu J Wang X Zhu H Gong P Wang T . Design, synthesis and biological evaluation of novel FXIa inhibitors featuring five-membered heterocycles as P2’ fragments. Eur J Med Chem. (2026) 303:118457. doi: 10.1016/j.ejmech.2025.118457 41401594

[138] Gailani D Gruber A . Factor XI as a therapeutic target. Arterioscler Thromb Vasc Biol. (2016) 36:1316–22. doi: 10.1161/atvbaha.116.306925 27174099 PMC4919154

[139] Muller F Renne T . Novel roles for factor XII-driven plasma contact activation system. Curr Opin Hematol. (2008) 15:516–21. doi: 10.1097/MOH.0b013e328309ec85 18695377

[140] Thomas D Kanefendt F Schwers S Unger S Yassen A Boxnick S . First evaluation of the safety, pharmacokinetics, and pharmacodynamics of BAY 2433334, a small molecule targeting coagulation factor XIa. J Thromb Haemost. (2021) 19:2407–16. doi: 10.1111/jth.15439 34192419 PMC8518835

[141] Kanefendt F Brase C Jungmann N Fricke R Engelen A Schmitz S . Pharmacokinetics of asundexian with combined CYP3A and P-gp inhibitors and an inducer: Target *in vitro* and *in vivo* studies. Br J Clin Pharmacol. (2024) 90:1036–49. doi: 10.1111/bcp.15981 38048692

[142] Perera V Wang Z Lubin S Christopher LJ Chen W Xu S . Effects of rifampin on the pharmacokinetics and pharmacodynamics of milvexian, a potent, selective, oral small molecule factor XIa inhibitor. Sci Rep. (2022) 12:22239. doi: 10.1038/s41598-022-25936-2 36564395 PMC9789074

[143] Gouin-Thibault I Lecompte T Lasne D . Anticoagulant drugs targeting factor XI/XIa and coagulation tests: We urgently need reliable pharmacodynamic data. J Thromb Haemost. (2025) 23:1464–8. doi: 10.1016/j.jtha.2025.02.025 40056986

[144] Ferri N Colombo E Corsini A . Drug-drug interactions of FXI inhibitors: Clinical relevance. Hematol Rep. (2024) 16:151–63. doi: 10.3390/hematolrep16010016 PMC10970023 38534886

[145] Ali AE Becker RC . Factor XI: Structure, function and therapeutic inhibition. J Thromb Thromb. (2024) 57:1315–28. doi: 10.1007/s11239-024-02972-5 38622277 PMC11645426

[146] Ades M Simard C Vanassche T Verhamme P Eikelboom J Mavrakanas TA . Factor XI inhibitors: Potential role in end-stage kidney disease. Semin Nephrol. (2023) 43:151484. doi: 10.1016/j.semnephrol.2023.151484 38272779

[147] Zhu Y Zhu L Wang X Jin H . RNA-based therapeutics: An overview and prospectus. Cell Death Dis. (2022) 13:644. doi: 10.1038/s41419-022-05075-2 35871216 PMC9308039

[148] Crooke ST Baker BF Crooke RM Liang XH . Antisense technology: An overview and prospectus. Nat Rev Drug Discov. (2021) 20:427–53. doi: 10.1038/s41573-021-00162-z 33762737

[149] Wolberg AS Rosendaal FR Weitz JI Jaffer IH Agnelli G Baglin T . Venous thrombosis. Nat Rev Dis Primers. (2015) 1:15006. doi: 10.1038/nrdp.2015.6 27189130

[150] Esmon CT . Basic mechanisms and pathogenesis of venous thrombosis. Blood Rev. (2009) 23:225–9. doi: 10.1016/j.blre.2009.07.002 19683659 PMC2762278

[151] Mackman N . New insights into the mechanisms of venous thrombosis. J Clin Invest. (2012) 122:2331–6. doi: 10.1172/jci60229 22751108 PMC3386811

[152] Budnik I Brill A . Immune factors in deep vein thrombosis initiation. Trends Immunol. (2018) 39:610–23. doi: 10.1016/j.it.2018.04.010 29776849 PMC6065414

[153] von Bruhl ML Stark K Steinhart A Chandraratne S Konrad I Lorenz M . Monocytes, neutrophils, and platelets cooperate to initiate and propagate venous thrombosis in mice *in vivo*. J Exp Med. (2012) 209:819–35. doi: 10.1084/jem.20112322 22451716 PMC3328366

[154] Fuchs TA Brill A Duerschmied D Schatzberg D Monestier M Myers DD Jr . Extracellular DNA traps promote thrombosis. Proc Natl Acad Sci USA. (2010) 107:15880–5. doi: 10.1073/pnas.1005743107 20798043 PMC2936604

[155] Heit JA . Epidemiology of venous thromboembolism. Nat Rev Cardiol. (2015) 12:464–74. doi: 10.1038/nrcardio.2015.83 26076949 PMC4624298

[156] Asada Y Yamashita A Sato Y Hatakeyama K . Pathophysiology of atherothrombosis: Mechanisms of thrombus formation on disrupted atherosclerotic plaques. Pathol Int. (2020) 70:309–22. doi: 10.1111/pin.12921 32166823 PMC7317428

[157] Yamashita A Asada Y . Underlying mechanisms of thrombus formation/growth in atherothrombosis and deep vein thrombosis. Pathol Int. (2023) 73:65–80. doi: 10.1111/pin.13305 36598039 PMC11551813

[158] Owens AP 3rd Mackman N . Role of tissue factor in atherothrombosis. Curr Atheroscler Rep. (2012) 14:394–401. doi: 10.1007/s11883-012-0269-5 22886473 PMC5886738

[159] Olie RH van der Meijden PEJ Ten Cate H . The coagulation system in atherothrombosis: Implications for new therapeutic strategies. Res Pract Thromb Haemost. (2018) 2:188–98. doi: 10.1002/rth2.12080 30046721 PMC6055505

[160] Rao SV O'Donoghue ML Ruel M Rab T Tamis-Holland JE Alexander JH . 2025 ACC/AHA/ACEP/NAEMSP/SCAI guideline for the management of patients with acute coronary syndromes: A report of the American College of Cardiology/American Heart Association Joint Committee on Clinical Practice Guidelines. Circulation. (2025) 151:e771–862. doi: 10.1161/cir.0000000000001309 40014670

[161] Kleindorfer DO Towfighi A Chaturvedi S Cockroft KM Gutierrez J Lombardi-Hill D . 2021 guideline for the prevention of stroke in patients with stroke and transient ischemic attack: A guideline from the American Heart Association/American Stroke Association. Stroke. (2021) 52:e364–467. doi: 10.1161/str.0000000000000375 34024117

[162] Cucchiara B Connors JM Rabinstein AA Sharma M . Antithrombotic therapy for noncardioembolic ischemic stroke: Current evidence and future directions. Stroke. (2026) 57:1439–49. doi: 10.1161/strokeaha.125.054846 41822978

[163] Al-Saady NM Obel OA Camm AJ . Left atrial appendage: structure, function, and role in thromboembolism. Heart. (1999) 82:547–54. doi: 10.1136/hrt.82.5.547 10525506 PMC1760793

[164] Ding WY Gupta D Lip GYH . Atrial fibrillation and the prothrombotic state: Revisiting Virchow's triad in 2020. Heart. (2020) 106:1463–8. doi: 10.1136/heartjnl-2020-316977 32675218

[165] Boeckh-Behrens T Kleine JF Zimmer C Neff F Scheipl F Pelisek J . Thrombus histology suggests cardioembolic cause in cryptogenic stroke. Stroke. (2016) 47:1864–71. doi: 10.1161/strokeaha.116.013105 27197854

[166] Khorana AA Mackman N Falanga A Pabinger I Noble S Ageno W . Cancer-associated venous thromboembolism. Nat Rev Dis Primers. (2022) 8:11. doi: 10.1038/s41572-022-00336-y 35177631

[167] Tatsumi K . The pathogenesis of cancer-associated thrombosis. Int J Hematol. (2024) 119:495–504. doi: 10.1007/s12185-024-03735-x 38421488

[168] Campello E Simioni P Prandoni P Ferri N . Clinical pharmacology of factor XI inhibitors: New therapeutic approaches for prevention of venous and arterial thrombotic disorders. J Clin Med. (2022) 11:6314. doi: 10.3390/jcm11216314 36362541 PMC9654315

[169] Galli M Laborante R Ortega-Paz L Franchi F Rollini F D'Amario D . Factor XI inhibitors in early clinical trials: A meta-analysis. Thromb Haemostasis. (2023) 123:576–84. doi: 10.1093/eurheartj/ehad655.2833 36841245

[170] Prakash TP Yu J Migawa MT Kinberger GA Wan WB Østergaard ME . Comprehensive structure–activity relationship of triantennaryN-acetylgalactosamine conjugated antisense oligonucleotides for targeted delivery to hepatocytes. J Med Chem. (2016) 59:2718–33. doi: 10.1021/acs.jmedchem.5b01948 26914862

[171] Vasquez G Freestone Graeme C Wan WB Low A De Hoyos CL Yu J . Site-specific incorporation of 5′-methyl DNA enhances the therapeutic profile of gapmer ASOs. Nucleic Acids Res. (2021) 49:1828–39. doi: 10.1093/nar/gkab047 33544849 PMC7913697

